# Novel Bioactive Natural Products from Marine-Derived *Penicillium* Fungi: A Review (2021–2023)

**DOI:** 10.3390/md22050191

**Published:** 2024-04-23

**Authors:** Fang Lv, Yanbo Zeng

**Affiliations:** 1 Beijing Key Laboratory for Separation and Analysis in Biomedicine and Pharmaceuticals, School of Life Science, Beijing Institute of Technology, Beijing 100081, China; lvfangbeijing@bit.edu.cn; 2 Hainan Provincial Key Laboratory for Functional Components Research and Utilization of Marine Bio-Resources & National Key Laboratory for Tropical Crop Breeding, Institute of Tropical Bioscience and Biotechnology, Chinese Academy of Tropical Agricultural Sciences, Haikou 571101, China

**Keywords:** marine-derived *Penicillium*, marine natural products, polyketides, antibacterial activity, cytotoxicity

## Abstract

Marine-derived *Penicillium* fungi are productive sources of structurally unique and diverse bioactive secondary metabolites, representing a hot topic in natural product research. This review describes structural diversity, bioactivities and statistical research of 452 new natural products from marine-derived *Penicillium* fungi covering 2021 to 2023. Sediments are the main sources of marine-derived *Penicillium* fungi for producing nearly 56% new natural products. Polyketides, alkaloids, and terpenoids displayed diverse biological activities and are the major contributors to antibacterial activity, cytotoxicity, anti-inflammatory and enzyme inhibitory capacities. Polyketides had higher proportions of new bioactive compounds in new compounds than other chemical classes. The characteristics of studies in recent years are presented.

## 1. Introduction

Marine-derived fungi have a variety of medical applications due to their capability of generating various enzymes and antimicrobial agents [1]. Since the first species *Sphaeria posidoniae* (*Halotthia posidoniae*) on the rhizome of the sea grass *Posidonia oceanica* was studied in 1846 [2], scientists have never stopped studying the natural products (NPs) of marine-derived fungi [3]. The rapid development of marine bio-technology and ever-increasing needs of clinic applications resulted in the emergence of marine natural products as alternative drug sources in the early 1990s [4] and the voluminous output in natural product research from the fungi isolated from different marine animals, seaweeds and sediments, with many new bioactive compounds being described each year [5].

As an essential part of marine micro-organisms, *Penicillium* fungi have received great attention among all marine-derived fungi, accounting for 22% of NPs of marine fungal origin, and play an important role in the discovery of marine natural products with bioactivities and novel structures [6]. Several reviews on natural products isolated from marine-derived *Penicillium* species have been published [4,6,7,8]. A total of 390 new secondary metabolites from marine-derived *Penicillium* fungi were highlighted from 1991 to 2014 [6], and 188 new secondary metabolites were summarized from 2015 to 2020 [7]. More than 200 cytotoxic or antitumor compounds isolated from marine *Penicillium* fungus were included from 1991 to 2017 [4]. Newly reported alkaloids produced by marine-derived *Penicillium* species were offered from 2014 to 2018 [8]. Recently, some remarkable achievements have been made in the study of marine-derived *Penicillium* fungi, including an MS/MS targeted molecular networking approach for the discovery of rare communesins [9], the investigation of the apoptosis mechanism of dicitrinone G from by the analyses of the protein–protein interaction (PPI) network and Western blot [10], and so on. These effective approaches have given rise to the generation of unique chemicals and huge biological diversity, making marine-derived *Penicillium* fungi a hotspot for utilization in the discovery of new drug leads.

A systematic review of the origins, structures, and bioactivities of 452 new NPs produced by marine-derived *Penicillium* species from January 2021 to December 2023 is provided in this review, based on 115 studies searching in the SciFinder database with marine-derived *Penicillium* as the key word, with English as the language. The review also covers fifty-one new marine natural products only described based on the HPLC-MS/MS analyses [9]; two previously reported marine natural products with significant new bioactivities [11,12], three known marine natural products supplied the structural NMR data [13,14,15], and two compounds were presented as new natural products [15,16], but their structures are not shown. In addition, due to the narrow publication timespan, seven pairs of new compounds possessing different structures were given the same trivial name, respectively. In this review, the first reported compounds were given the suffix a [17,18,19], and the other ones were given the suffix b [20,21].

## 2. New Bioactive Compounds from Marine-Derived *Penicillium* Fungi

### 2.1. Polyketides

#### 2.1.1. Azaphilones

**Figure 1:**
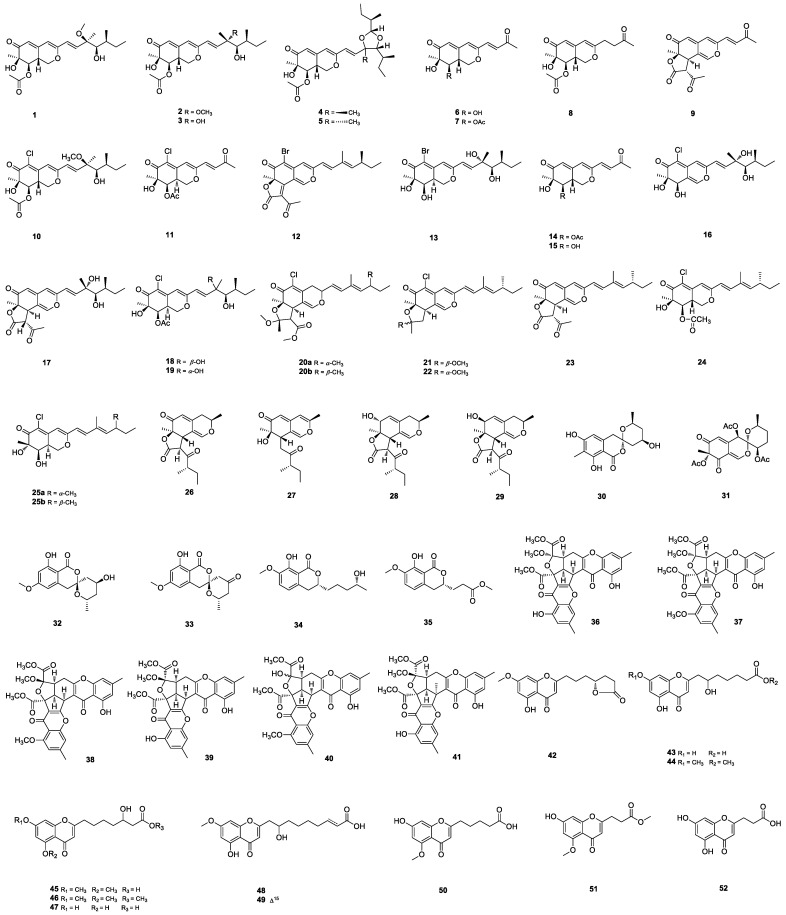
Structures of **1**–**52**.

Azaphilones are a class of structurally diverse fungal metabolites that are mainly defined as polyketides possessing a highly oxygenated pyranoquinone bicyclic core and a quaternary carbon center [17]. A series of azaphilones with novel structures and remarkable bioactivities were reported from marine-derived *Penicillium* fungi. Penicil-azaphilones Ia–N (**1**, **2** and **6**–**9**), *epi*-geumsanol D (**3**) and penidioxolanes C (**4**) and D (**5**) (Figure 1) were isolated from the sponge-derived *P. sclerotiorum* E23Y-1A culture [17]. Penicil-azaphilone Ia-N **9** showed moderate anti-inflammatory activity with an IC_50_ value of 22.63 ± 2.95 μM, whereas **4** exhibited various cytotoxic activities. The same strain produced two chlorinated azaphilones, penicilazaphilones F (**10**) and G (**11**) (Figure 1), with a moderate anti-inflammatory effect [22]. Based on a one strain–many compounds (OSMAC) approach, two new brominated analogs, 5-bromoisorotiorin (**12**) and penicilazaphilone Ha (**13**) (Figure 1), were obtained from *P. sclerotiorum* E23Y-1A by the addition of NaBr into the culture medium. Both showed moderate antibacterial activities against *Staphylococcus aureus* ATCC 25923 with inhibition zone diameters of 8.08 ± 0.01 and 7.50 ± 0.05 mm, respectively [18]. New azaphilones, penicilazaphilones Hb–Ib (**14**–**15**), 11-*epi*-geumsanols B and F (**17**–**16**), 8a-*epi*-hypocrellone A (**18**), and 8a-*epi*-eupenicilazaphilone C (**19**) (Figure 1), were isolated from algae-derived *P. sclerotiorum* [20,23]. Azaphilone **19** significantly promoted SMAD-mediated transcriptional activities stimulated by TGF-*β* [23]. Azaphilone *E/Z* isomers isochromophilone H (**20a**/**b**), sclerotiorins A (**21**) and B (**22**), ochlephilone (**23**), isochromophilone IV (**24**), and isochromophilone J (**25a**/**b**) (Figure 1) were isolated from the culture broth of the mangrove-derived fungus *P. sclerotiorum* HY5. Azaphilones **22** and **23** exhibited potent phytotoxicity against the growth of radicles and plumules on *Amaranthus retroflexus* L., with EC_50_ values ranging from 234.87 to 320.84 μM, compared to the positive control glufosinate-ammonium, with EC_50_ values of 555.11 μM for radicles, and 656.04 μM for plumules [24]. Chermesinones D–G (**26**–**29**) (Figure 1) were isolated from marine-derived *P. chermesinum* FS625 [25]. Daldinins G-H (**30**–**31**) (Figure 1) were isolated from the soft-coral-derived *P. glabrum* glmu 003 [26].

#### 2.1.2. Isocoumarins

Penicillols A (**32**) and B (**33**) (Figure 1) featuring spiroketal rings were isolated from the mangrove-derived *Penicillium* sp. BJR-P2. Pannicillol B (**33**) exhibited significant inhibitory activity on NO production with an IC_50_ value of 12 μM [27]. Peniciisocoumarins I (**34**) and J (**35**) (Figure 1) were obtained from the mangrove-derived *Penicillium* sp. GXIMD 03001 [28].

#### 2.1.3. Chromones

The marine-derived *P. citrinum* BCRC 09F458 yielded a class of rare chromone derivatives, epiremisporines C–H (**36**–**41**) (Figure 1). Epiremisporines **37** and **38** could significantly inhibit fMLP-induced superoxide anion generation, with IC_50_ values ≤ 8.28 μM. Through the mitochondrial- and caspase 3-dependent pathways, **38** and **41** markedly induced the apoptosis of A549 with IC_50_ values of 43.82 ± 6.33 and 31.43 ± 3.01 μM, respectively. Furthermore, **41** obviously induced apoptosis of HT-29 cells, via Bcl-2, Bax, and caspase 3 signaling cascades [29,30]. Eleven 5,7-dioxygenated chromones penithochromones M–W (**42**–**52**) (Figure 1), bearing an aliphatic acid side chain, were isolated from the deep-sea-sediment-derived *P. thomii* YPGA3 [31,32]. Penithochromones **47**–**49** exhibited remarkable inhibition against *α*-glucosidase with IC_50_ values ranging from 842 to 1017 μM, which are more active than the positive control acarbose [31].

#### 2.1.4. Citrinins

**Figure 2:**
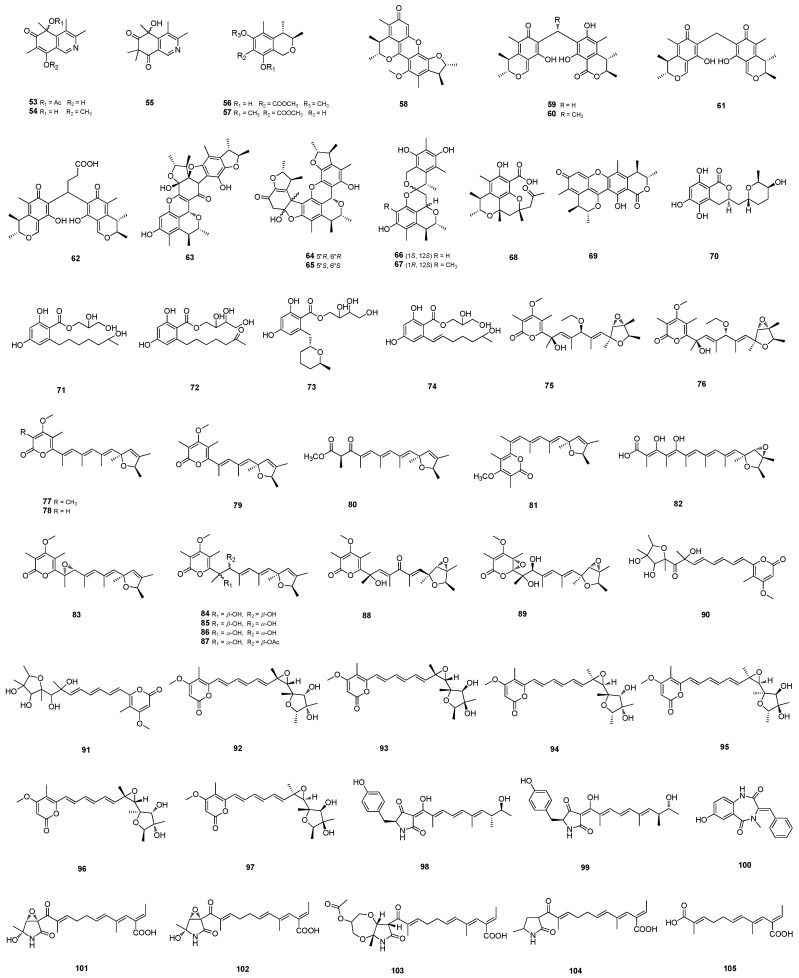
Structures of **53**–**105**.

Citrinins, as an important class of polyketide mycotoxins, usually have core structure skeletons like benzopyran, benzofuran, and quinone-pyran, etc. [33]. Three extremely rare nitrogen-containing citrinin derivatives, isoquinocitrinins B–D (**53**–**55**), and their corresponding enantiomers (**53a**/**b**, **54a**/**b**, **55a**/**b**) (Figure 2) were acquired from the hydrothermal-vent-sediment-derived *Penicillium* sp. TW131-64. These products exhibited potential anti-*H. pylori* activities towards the standard strain and multidrug-resistant clinical isolates with MIC values ranging from 0.25 to 8 μg/mL, indicating a comparable or even better killing activity than metronidazole. Citrinin derivatives (3*R*,4*S*)-8-hydroxy-6-methoxy-3,4,5-trimethylisochromane-7-carboxylatemethyl (**56**), (3*R*,4*S*)-6-hydroxy-8-methoxy -3,4,5-trimethylisochromane-7-carboxy latemethyl (**57**), and penicitrinone J (**58**) (Figure 2) were also obtained from this strain [33]. Rare carbon-bridged citrinin dimers, dicitrinones G–J (**59**–**62**) (Figure 2) were isolated from starfish-derived *Penicillium* sp. GGF 16-1-2 and exhibited strong antifungal activities against *Colletotrichum gloeosporioides* with LD_50_ values ranging from 9.58 μg/mL to 16.14 μg/mL. Furthermore, **59** showed significant cytotoxicity against human pancreatic cancer cell lines BXPC-3 and PANC-1, which could induce apoptosis by activating caspase 3 proteins (CASP3) [10]. Neotricitrinols A–C (**63**–**65**) (Figure 2), isolated from the marine-sediment-derived *P. citrinum* W23, feature a unique octacyclic carbon scaffold among the few reported citrinin trimers. Neotricitrinol **64** showed potential anti-osteoporosis activity by promoting osteoblastogenesis and inhibiting adipogenic differentiation on primary bone mesenchymal stem cells [34]. Xerucitrinins B and C (**66**–**67**) (Figure 2) bearing a 6,6-spiroketal moiety were isolated from hydrothermal vent-associated *P. citrinum* Y34 [35]. Penicitrinol P (**68**) and dicitrinol D (**69**) (Figure 2) were isolated from the sponge-derived *Penicillium* sp. SCSIO 41302 [36] and *Penicillium* sp. SCSIO41303, respectively [37].

#### 2.1.5. *β*-Resorcylic Acid

Resorcylic acid lactones (RALs) are structurally diverse polyketides, which usually consist of condensed resorcylic and macrolide cycles, sometimes possessing an open macrolide cycle [38]. Five *β*-resorcylic acid derivatives, 14-hydroxyasperentin B (**70**), *β*-resoantarctines A–C (**71**–**73**) (Figure 2) and 8-dehydro-*β*-resoantarctine A (**74**) (Figure 2), were isolated from the brown-alga-derived *P. antarcticum* KMM 4685. *β*-resorcylic acid derivatives **71**–**72** and **74** (Figure 2) inhibited the activity of *p*-glycoprotein at their noncytotoxic concentrations and consequently synergized with docetaxel in *p*-glycoprotein-overexpressing drug-resistant cells [38].

#### 2.1.6. Verrucosidin

Verrucosidins belong to a family of highly reduced polyketides, generally sharing a methylated *α*-pyrone, a conjugated polyene linker, and an epoxidated tetrahydrofuran ring [39]. A pair of epimers, 9-*O*-ethylpenicyrones A (**75**) and B (**76**) (Figure 2), were isolated and identified from the marine-sediment-derived *P. cyclopium* SD-413. Epimers **75** and **76** showed antibiotic activity against aquatic pathogen *A. hydrophilia*, each with an MIC value of 8 μg/mL [40]. Poloncosidins A–K (**77**–**87**) (Figure 2) [39,41] were identified from the cold-seep-sediment-derived *P. polonicum* CS-252. Poloncosidins **77**–**86** were the first verrucosidins with a 2,5-dihydrofuran ring. Most of these compounds exhibited inhibitory activities against several human and aquatic pathogens with MIC values ranging from 4 to 32 μg/mL. Verrucosidinols A (**88**) and B (**89**) (Figure 2) were isolated from the marine-sediment-derived *P. griseofulvum* MCCC 3A00225 [42].

#### 2.1.7. Citreoviridins

Citreoviridins H (**90**) and I (**91**) (Figure 2) were isolated from the mangrove-derived *Penicillium* sp. BJR-P2 [27]. Citreoviridins J-O (**92**–**97**) (Figure 2) belonged to diastereomers of 6,7-epoxycitreoviridin with different chiral centers at C-2–C-7 and were isolated from the deep-sea-sediment-derived *P. citreonigrum* MCCC 3A00169 [43].

#### 2.1.8. Nitrogen-Containing Polyketides

Fungal polyketide–amino acid hybrids are a large family of secondary metabolites produced by PKS-NRPS assembly lines [44]. The derivatives oxopyrrolidine A (**98**) and B (**99**) (Figure 2) were isolated based on bioactivity screening and chemical profiles from the marine-derived *P. oxalicum* MEFC104 [44]. 7-hydroxy-3,10-dehydrocyclopeptine (**100**) (Figure 2) was isolated from the mangrove-sediment-derived *P. polonicum* MCCC3A 00951 [45]. Fusarin derivatives steckfusarins A–E (**101**–**105**) (Figure 2) were isolated from the green-algae-derived *P. steckii* SCSIO41040. Steckfusarin A (**101**) exhibited antioxidant activity against DPPH, with an IC_50_ value of 74.5 μg/mL [46].

#### 2.1.9. Sorbicillinoids

**Figure 3:**
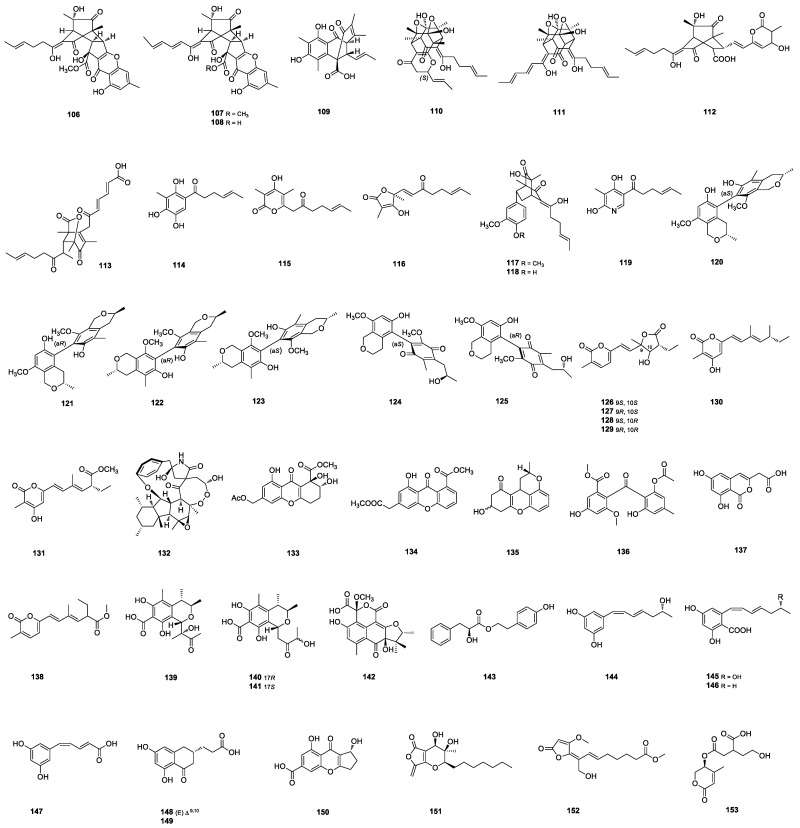
Structures of **106**–**153**.

Bisorbicillchaetones A-C (**106**–**108**) (Figure 3) were the first examples of hybrid sorbicillinoids containing a coniochaetone unit and isolated from the deep-sea-sediment-derived *Penicillium* sp. SCSIO06868. Bisorbicillchaetones **106** and **107** exhibited moderate inhibitory effects on NO production in lipopolysaccharide (LPS)-activated RAW264.7 cells with IC_50_ values of 80.3 ± 3.6 μM and 38.4 ± 3.3 μM, respectively [47]. Various sorbicillinoids, including two hybrid sorbicillinoids, 10-methylsorbiterrin A (**109**) and dihydrotrichodermolidic acid (**113**); three bisorbicillinoids, epitetrahydrotrichodimer ether (**110**), demethyldihydro-trichodimerol (**111**) and bisorbicillpyrone A (**112**); and three monomeric sorbicillinoids, 5-hydroxy-dihydrodemethlsorbicillin (**114**), sorbicillpyrone A (**115**) and 5,6-dihydrovert-inolide (**116**) (Figure 3); were isolated from the deep-sea-sediment-derived *Penicillium* sp. SCSIO06871. Monomeric sorbicillinoid **114** displayed more potent inhibitory activity against α-glycosidase than acarbose with an IC_50_ value of 36.0 μM [48]. Sorbicatechols C (**117**) and D (**118**) (Figure 3) were isolated from deep-sea-derived *P. allii-sativi* MCCC3A00580. Sorbicatechol D (**118**) inhibited HT-29 cells in a dose-dependent manner [49]. A sorbicillinoid, (4E)-1-(4,6-dihydroxy-5-methylpyridin-3-yl)hex-4-en-1-one (**119**) (Figure 3), was isolated from the mangrove-derived *Penicillium* sp. DM815 [50].

#### 2.1.10. Isochromans

Penicisteckins A–F (**120**–**125**) (Figure 3) represented novel biaryl scaffolds containing both central and axial chirality elements and isolated from the beach-mud-derived *P. steckii* HNNU-5B18 [51].

#### 2.1.11. *α*-Pyrone Polyketides

Six *α*-pyrone polyketides, penipyrols C–G (**126**–**130**) (Figure 3) and methyl-penipyrol A (**131**), were isolated from the mangrove-derived *Penicillium* sp. HDN-11-131. Penipyrols **127**–**129** possess a rare skeleton featuring *γ*-butyrolactone linked to an *α*-pyrone ring through a double bond. Penipyrol **127** can induce pancreatic *β*-cell regeneration in zebrafish at 10 μM, demonstrating promising anti-diabetes potential [52].

#### 2.1.12. Hirsutellones 

The natural hirsutellones are made of a decahydrofluorene polyketide core (rings A, B and C) involved in a highly strained 12- or 13-membered para-cyclophane (ring D) and highly functionalized 5-hydroxypyrrolidinone [53]. Perpyrrospirone A (**132**) (Figure 3) was the first example of hirsutellone peroxide from the marine-derived *P. citrinum*, and characterized an unprecedented 6/5/6/8/5/13/6 oxahexacyclic scaffold with a unique peroxide-bridged 8,9-dioxa-2-azaspiro[4.7] dodecane core [53].

#### 2.1.13. Xanthones and Benzophenones

Xanthones, known as 9H-xanthen-9-ones, are a class of yellow compounds bearing a dibenzo-*γ*-pyrone scaffold, which are often regarded as privileged structures for binding with a variety of targets [54]. Tetrahydroxanthone 11-*O*-acetylaspergillusone B (**133**) and the fully aromatic xanthone 7-dehydroxyhuperxanthone A (**134**) (Figure 3) were isolated from the deep-sea-sediment-derived *Penicillium* sp. MCCC 3A00126 [54]. Penicixanthene E (**135**) (Figure 3), the first reported xanthene derivative in which a carbon–carbon double bond was reduced, was isolated from the mangrove-derived *Penicillium* sp. GXIMD 03101 [55]. Benzophenone derivative penibenzophenone C (**136**) (Figure 3) and a new natural product, penibenzophenone D, were isolated from the mangrove-derived *Penicillium* sp. Penibenzophenone C (**136**) showed moderate antibacterial activity against methicillin-resistant *S. aureus* with an MIC value of 3.12 μg/mL [16].

#### 2.1.14. Hydroxybenzenes

Peniketide A (**137**) and a methyl ester of penipyrol A (**138**) (Figure 3) were isolated from the marine-sediment-derived *Penicillium* sp. SCZ-1. Peniketide A **137** bearing a two-carbon side chain at C-2 is seldom found among natural isocoumarins [56]. The deep-sea-sediment-derived *P. citrinum* W17 yielded penidihydrocitrinins A–C (**139**–**141**) (Figure 3). Three isolates exhibited significant inhibitory effects on LPS-stimulated nitric oxide (NO) production in murine brain microglial BV-2 cells in a dose–response manner [57]. Peniciphenalenin G (**142**) (Figure 3) was isolated from the marine-derived *P. oxalicum* [58]. Penicinone C (**143**) (Figure 3) was identified from the mangrove-derived *Penicillium* sp. LA032 [59]. Six new polyketide derivatives (**144**–**149**) (Figure 3) were isolated from the hydrothermal-vent-sediment-derived *Penicillium* sp. TW58-16. Polyketide derivatives **144**–**147** showed strong *a*-glucosidase inhibitory effects with inhibition rates of 73.2%, 55.6%, 74.4%, and 32.0%, respectively, which were comparable with or even better than that of acarbose, a known *α*-glucosidase inhibitor [60]. Coniochaetone N (**150**) (Figure 3) was isolated from the deep-sea-sediment-derived *Penicillium* sp. SCSIO06868 [61].

#### 2.1.15. Lactones

Penicinones A (**151**) and B (**152**) (Figure 3) were identified from the mangrove-derived *Penicillium* sp. LA032. Penicinone A **151**, a rare furo[3,4-b]pyran-5-one skeleton with an *n*-heptyl moiety, was identified and found to exhibit significantly cytotoxic activity against the HepG2 cells, with an IC_50_ value of 3.87 ± 0.74 μM [59]. Walterolactone E (**153**) (Figure 3) was isolated from the hydrothermal-vent-sediment-derived *Penicillium* sp. TW58-16 [62].

#### 2.1.16. Olefinic Acids and Their Derivatives

**Figure 4:**
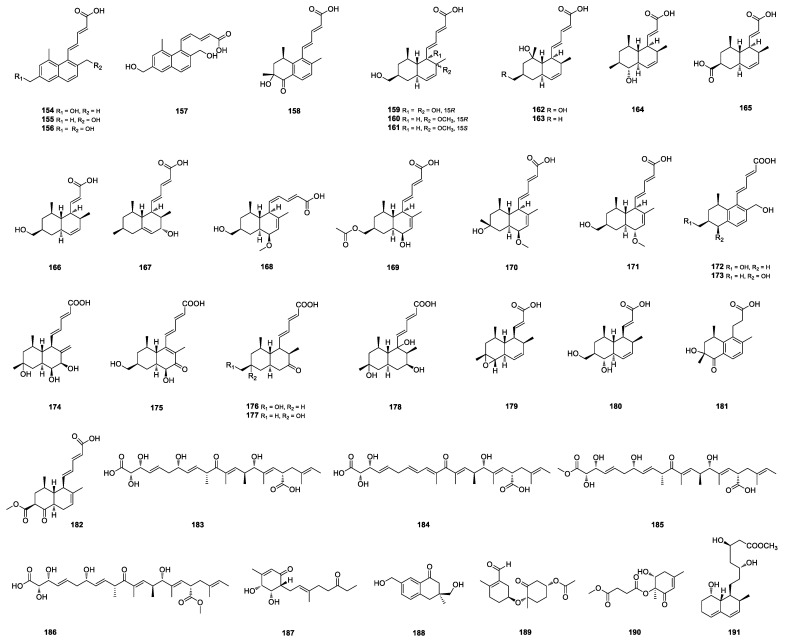
Structures of **154**–**191**.

Tanzawaic acids are a small class of polyketides, characterized by a trans-decalin (A/B fusion) scaffold, isolated mainly from the genus *Penicillium* [63]. The coral-derived *P. steckii* AS-324 yielded a series of tanzawaic acids, including steckwaic acids A–D (**154**–**157**), 11-ketotanzawaic acid D (**158**), 6,15-dihydroxytanzawaic acid M (**159**), 15*R*-methoxy-tanzawaic acid M (**160**), 15*S*-methoxytanzawaic acid M (**161**), 8-hydroxytanzawaic acid M (**162**), and 8-hydroxytanzawaic acid B (**163**) [64], steckwaic acids Ea-Ia (**164**–**168**), 18-*O*-acetyltanzawaic acid R (**169**), 10-hydroxytanzawaic acid U (**170**), and 13*R*-tanzawaic acid S (**171**) [19] (Figure 4). Among them, **171** showed potent activity against *Escherichia coli* with an MIC value of 8 μg/mL. Steckwaic acids Eb-Ib (**172**–**176**) and J-K (**177**–**178**) (Figure 4) were isolated from the green-algae-derived *P. steckii* SCSIO 41040. Steckwaic acid **173** inhibited LPS-induced nuclear factor kappa-B (NF-*κ*B) with an IC_50_ value of 10.4 μM, which is the first report of osteoclastogenesis inhibitory activity for tanzawaic acid derivatives [63]. Penicisteck acids A–D (**179**–**182**) (Figure 4) were isolated from the mangrove-derived *P. steckii* SCSIO 41025. Penicisteck acids **179**–**181** were highly oxygenated decalin derivatives harboring an unusual propanoic acid unit at C-1 [65]. Penifellutins A (**183**) and B (**184**), possessing a 22 carbons linear skeleton, were isolated from the co-culture of the deep-sea-derived fungi *P. crustosum* PRB-2 and *P. fellutanum* HDN14-323 along with two esterification products, penifellutins C (**185**) and D (**186**) (Figure 4). Penifellutins A (**183**) and B (**184**) showed obvious inhibitory activity on the liver hyperplasia of zebrafish larvae at a concentration of 10 μmol/L, while penifellutins C (**185**) and D (**186**) showed no activity, indicating that two carboxyls in the structure were important active sites [66].

#### 2.1.17. Other Polyketides

Rubenpolyketone A (**187**) (Figure 4) featuring cyclohexenone condensed with a methyl octenone chain was identified from the Magellan Seamount-derived *P. rubens* AS-130 [67]. Oxalichroman A (**188**) and oxalihexane A (**189**) (Figure 4) were isolated from the red algae-derived *P. oxalicum* 2021CDF-3. Oxalihexane A (**189**), formed by a cyclohexane and cyclohexanone moiety via an ether bond, showed a remarkable inhibitory effect on the human pancreatic cancer PATU8988T cell line through downregulation of the expression level of cyclin D1 [68]. Leptosphaerone D (**190**) (Figure 4) was isolated from the hydrothermal-vent-sediment-derived *Penicillium* sp. TW58-16 [62]. 15-*O*-methyl ML-236A (**191**) (Figure 4) was isolated from the deep-sea-sediment-derived *P. solitum* MCCC 3A00215 [69].

### 2.2. Alkaloids

#### 2.2.1. Indoles

**Figure 5:**
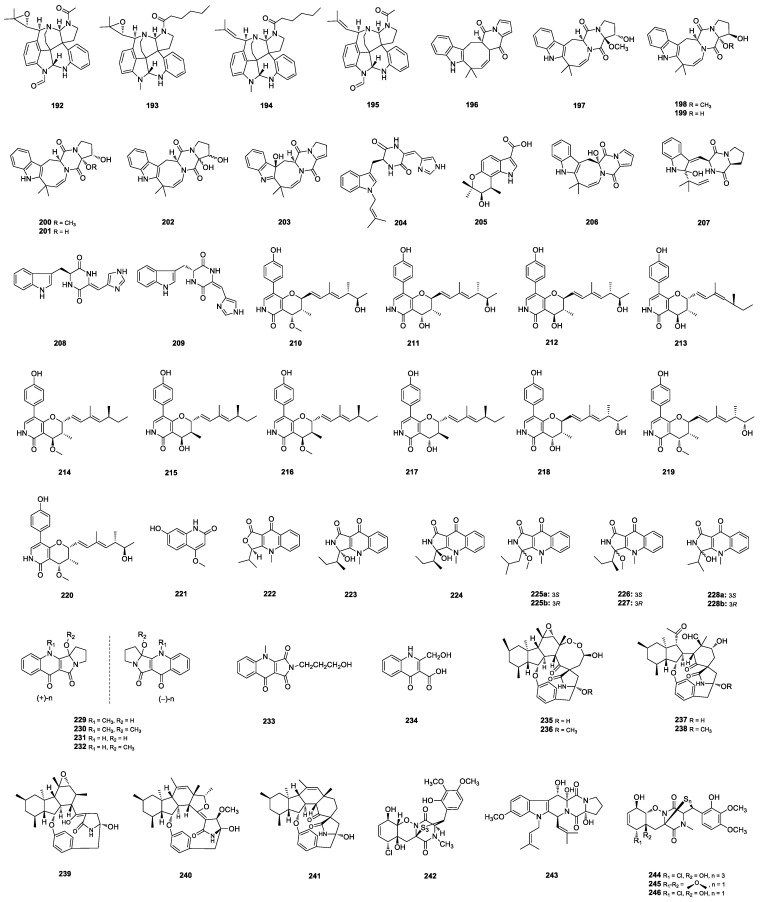
Structures of **192**–**246**.

Communesins are a class of complex indole alkaloids isolated from the *Penicillium* fungi. The marine-sediment-derived *P. expansum* was studied using a targeted molecular networking approach, allowing the detection of 55 new communesins. Among of them, communesins M-P (**192**–**195**) (Figure 5) were isolated and showed moderate cytotoxicity against KB and MCF-7 human cancer cell lines in comparison to the positive control docetaxel [9]. The coral-derived *P. dimorphosporum* KMM 4689 yielded the very first deoxyisoaustamide alkaloid deoxy-14,15-dehydroisoaustamide (**196**) [70] and seven deoxyisoaustamide derivatives (**197**–**203**) [71] (Figure 5). Deoxyisoaustamide derivatives **199**, **201** and **202** (Figure 5) showed a statistical increase in paraquat-treated Neuro-2a cell viability by 30–39% at a concentration of 1 μM. Penilline D (**204**) (Figure 5) was isolated from the Antarctic fungus *Penicillium* sp. SCSIO 05705 [72]. Penindolacid A (**205**) (Figure 5) was isolated from the marine-sediment-derived *Penicillium* sp. LW92 [73]. Prenylated indole diketopiperazine alkaloids (PIDAs) penicamides A (**206**) and B (**207**) (Figure 5) were identified from the mangrove-derived *Penicillium* sp. LA032. Penicamide A (**206**) was the first example of PIDAs featuring a 6/5/8/6/5 pentacyclic ring system with an *α*-hydroxy group at C-11[59]. 11*S*-(−)-penilloid A (**208**) and 11*R*,14*E*-(+)-penilloid A (**209**) (Figure 5) were isolated from the marine-mud-derived *Penicillium* sp. ZZ1750 [74].

#### 2.2.2. Pyridones

Eleven new pyridone alkaloids, penicipyridones A-K (**210**–**220**) (Figure 5), were isolated from the marine-derived *P. oxalicum* QDU1. Penicipyridones **210**, **213**–**214**, **217** and **219**–**220** exhibited moderate inhibitory effects on NO production in the LPS-induced RAW264.7 macrophages, with IC_50_ values ranging from 9.2 to 19 μM [75].

#### 2.2.3. Quinolinones

Penicinolone (**221**) (Figure 5) was isolated from the sponge-derived *Penicillium* sp. SCSIO41033 [76]. AChE-inhibitory-activity-guided studies on the mangrove-derived *P. citrinum* YX-002 led to the isolation of quinolactone A (**222**), quinolactacin C1 (**223**), and 3-*epi*-quinolactacin C1 (**224**) (Figure 5). Quinolactone A (**222**) showed moderate AChE inhibitory activity with an IC_50_ value of 27.6 μmol/L [77]. *N*-methyl-4-quinolones quinolactacin E (**225a**/**b**), quinolactacins F1–F2 (**226**–**227**) and quinolactacin G (**228a**/**b**) (Figure 5) were isolated from the sponge-derived *Penicillium* sp. SCSIO 41303. Quinolactacin **226** exhibited enzyme inhibition activity against PL with an IC_50_ value of 24.6 μg/mL [37]. Four racemic mixtures, (±)-oxypenicinolines A–D (**229**–**232**), along with penicinolines F (**233**) and G (**234**) (Figure 5) were isolated from the mangrove-derived *P. steckii* SCSIO 41025. Racemic mixtures **229**–**232** shared an unusual 6/6/5/5 tetracyclic system incorporating a rare tetrahydro-pyrrolyl moiety, while **229** displayed *α*-glucosidase inhibitory activity with an IC_50_ value of 317.8 μM, which was more potent than that of acarbose (461.0 μM) [78].

#### 2.2.4. Decahydrofluorene-Class Alkaloids

Pyrrospirones K–Q (**235**–**241**) (Figure 5) were isolated from the soft-coral-derived *Penicillium* sp. SCSIO 41512. Pyrrospirones **235** and **237** possessed a novel decahydrofluorene-class alkaloid skeleton with 6/5/6/8/5/6/13 and 6/5/6/5/6/13 polycyclic systems, respectively. Pyrrospirones **235**–**237** and **239** showed antibacterial activity against all or some of the six pathogens *B. amyloliquefaciens*, *B. subtilis*, *E. coli*, *S. aureus*, *S. aureus* MRSA, and *S. agalactiae*. Pyrrospirones **238** and **240** displayed mild inhibitory activity against several PTPs with IC_50_ values of 39.4–100 μM [79].

#### 2.2.5. Piperazines

A trithiodiketopiperazine derivative, adametizine C (**242**) (Figure 5), was isolated from the mangrove-sediment-derived *P. ludwigii* SCSIO 41408. Adametizine C (**242**) showed cytotoxicity against prostate cancer cell line 22Rv1 with an IC_50_ value of 13.9 μM, and the strongest inhibitory activity against RANKL-induced osteoclast differentiation in bone marrow macrophage cells with 10 μM [80]. A diketopiperazine alkaloid, (8*S*,9*R*,12*R*,18*S*)-12-hydroxyl-fumitremorgin B (**243**) (Figure 5), was isolated from the hydrothermal-vent-sediment-derived *Penicillium* sp. TW58-16 [62]. Three epithiodiketopiperazine alkaloids, penigainamides A–C (**244**–**246**) (Figure 5), were isolated from the marine-derived *P. steckii* YE [81].

#### 2.2.6. Tetramic-Acid-Based Alkaloids

**Figure 6:**
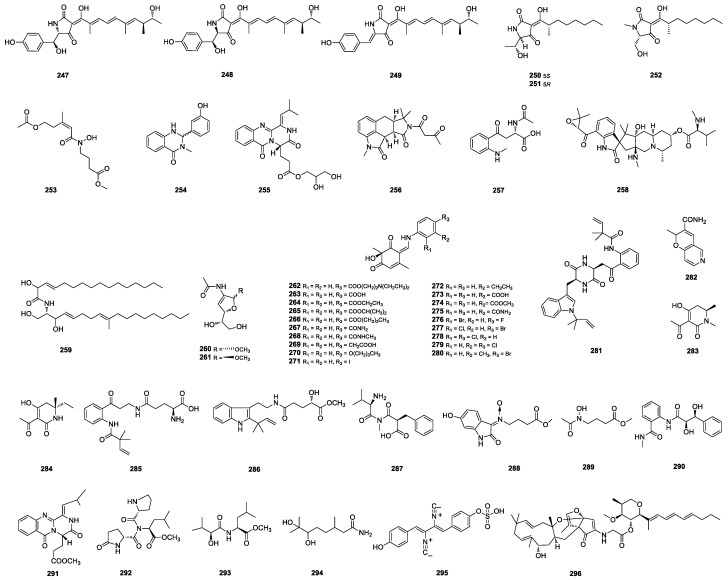
Structures of **247**–**296**.

Tolypocladenols D-F (**247**–**249**) (Figure 6) were isolated from the fresh and healthy leaves of the Apocynum venetum-derived fungus *P. oxalicum* QDU1. Tolypocladenol **249** exhibited moderate inhibitory effects on NO production in the LPS-induced RAW264.7 macrophages, with an IC_50_ value of 14 ± 1 μM [75]. Penicillenols G1–G2 (**250**–**251**) and H (**252**) (Figure 6) were isolated from cultures of the deep-sea-sediment-derived *Penicillium* sp. SCSIO06868. Penicillenol H (**252**) exhibited potent inhibitory activities against *S. aureus* and methicillin-resistant *S. aureus* with MIC values of both 2.5 mg/mL [61].

#### 2.2.7. Amines and Amides

(*Z*)-4-(5-acetoxy-*N*-hydroxy-3-methylpent-2-enamido) butanoate (**253**) (Figure 6) was isolated from the mangrove-derived *P. oxalicum* HLLG-13 and showed significant growth inhibition activities against newly hatched *Helicoverpa armigera* Hubner larvae, with an IC_50_ value of 200 μg/mL [15]. Polonimides E (**254**) and D (**255**) (Figure 6) were isolated from the sponge-derived *Penicillium* sp. SCSIO 41413 [82]. Speradine I (**256**) (Figure 6) was isolated from the soft-coral-derived *Penicillium* sp. SCSIO 41038 [83]. (*S*)-2-acetamido-4-(2-(methylamino)phenyl)-4-oxobutanoic acid (**257**) (Figure 6) was isolated from the deep-sea-gammarid-shrimp-derived *P. citrinum* XIA-16 [84]. A pentacyclic alkaloid, citrinadin C (**258**) (Figure 6), was isolated from the deep-sea-sediment-derived *P. citrinum* and showed cytotoxic activity against human liver cancer cell line MHCC97H, with an IC_50_ value of 16.7 μM [85]. (2*S*,2′*R*,3*R*,3′*E*,4*E*,8*E*)-*N*-2′-hydroxyhexadecanoyl-2-amino -9-methyl-4,8-octadecadiene-1,3-diol (**259**) (Figure 6), a ceramide, was isolated from the seawater-derived *P. chrysogenum* Y20-2. (2*S*,2′*R*,3*R*,3′*E*,4*E*,8*E*)-*N*-2′-hydroxyhexadecanoyl-2-amino -9-methyl-4,8-octadecadiene-1,3-diol (**259**) showed no pro-angiogenic activity using a zebrafish model [86]. *N*-acetyl-d-glucosamines penichryfurans A (**260**) and B (**261**) (Figure 6) were isolated from the red-alga-derived *P. chrysogenum*. Penichryfuran A (**260**) exhibited strong cytotoxicity against the HepG2 cell line with an IC_50_ value of 9.0 μM [87]. Talaroenamines F1–F19 (**262**–**280**) (Figure 6) were isolated from the wetland-derived *P. malacosphaerulum* HPU-J01 using a one-pot/two-stage precursor-directed biosynthesis approach. Talaroenamine **275** was cytotoxic against the K562 cell line with an IC_50_ value of 2.2 μM [88]. Peniokaramine (**281**) and penipyranopyridine (**282**) (Figure 6) were isolated from the hydrothermal-vent-sediment-derived *Penicillium* sp. LSH-3-1. Peniokaramine (**281)** showed moderate cytotoxic activity against A549 cells with an inhibition percentage of 53.43 ± 5.89% [89]. Penicidihydropyridones A (**283**) and B (**284**) (Figure 6) were isolated from the sponge-derived *Penicillium* sp. B9. Both of them intriguingly appeared to perturb PD-L1/PD-1 interactions with a considerable inhibitory rate of 88.40% for **283** and 70.72% for **284** with a concentration of 10 μg/mL [90]. (+)-solitumidine D (**285**) and (±)-solitumidine E (**286**) were isolated from the marine-sediment-derived *P. solitum* MCCC 3A00215 [69]. Penicmariae-crucis C acid (**287**), *N*-(6-hydroxy-2-oxoindolin-3-ylidene)-5′-methoxy-5′-oxobutyl-amine oxide (**288**), and methyl-1′-(*N*-hydroxyacetamido)-butanoate (**289**) (Figure 6) were isolated from the mangrove-derived *P. steckii* SCSIO 41025 [65]. Penigrisamide (**290**), aurantiomoate C (**291**), *N*,*N*-pyroglutamylleucinmethylester (**292**), methyl-2*S*-hydroxy-3-methylbutanoyl-L-leucinate (**293**), and 6*R*,7-dihydroxy-3,7-dimethyloctanamide (**294**) (Figure 6) were isolated from the marine-sediment-derived *P. griseofulvum* MCCC 3A00225 [42].

#### 2.2.8. Other Alkaloids

Sulfoxanthocillin (**295**) (Figure 6) was isolated from the deep-sea-sediment-derived *Penicillium* sp. SCSIO sof101. Sulfoxanthocillin (**295**) showed significant activity against series pathogens with MIC values ranging from 0.06 to 8.0 μg/mL and relatively low cytotoxicity against human tumor cell lines [91]. Penipyridinone B (**296**) (Figure 6) was isolated from the marine-mud-derived *Penicillium* sp. ZZ1750. Penipyridinone B (**296**) represented the first example of its structural type and showed potent antiglioma activity, with IC_50_ values of 2.45 μM for U87MG cells and 11.40 μM for U251 cells [74].

### 2.3. Terpenoids

#### 2.3.1. Sesquiterpenes

**Figure 7:**
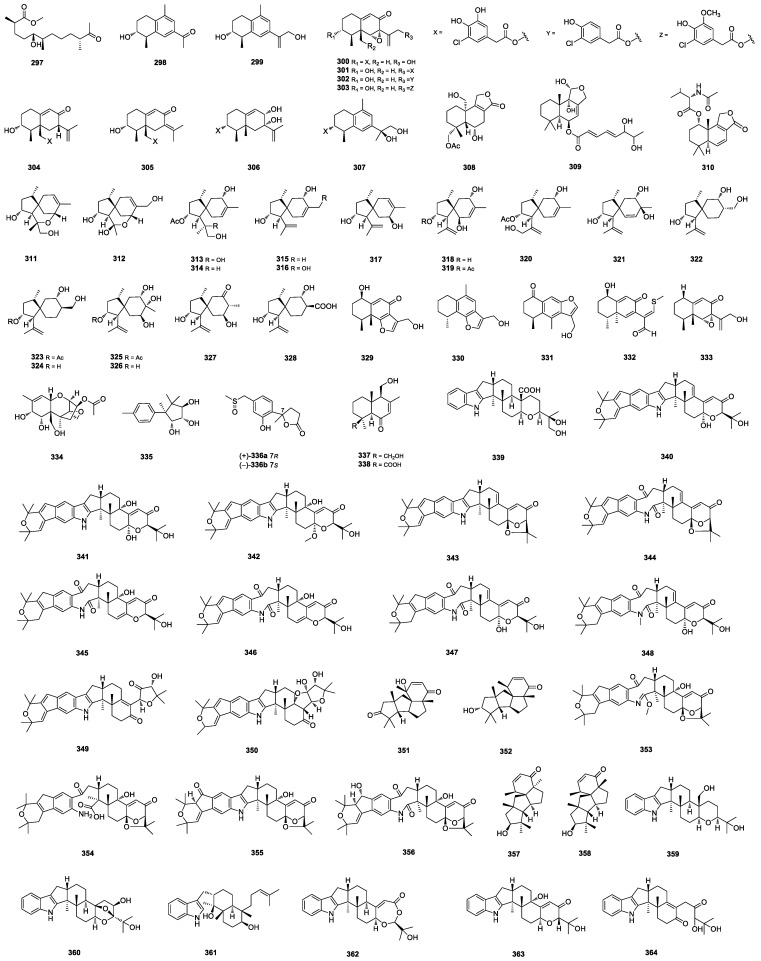
Structures of **297**–**364**.

A linear sesquiterpenoid, chermesiterpenoid D (**297**) (Figure 7), was identified from the Magellan Seamount-derived *P. rubens* AS-130 [67]. A series of eremophilane-type sesquiterpenes, copteremophilanes A–J (**298**–**307**) (Figure 7), were isolated from the marine-sponge-derived *P. copticola*. Analogs **298**, **299**, and **307** represented a group of uncommon skeletons of eremophilanes with an aromatic ring and a methyl migration from C-5 to C-9. The incorporation of a chlorinated phenylacetic unit in **300**–**306** has rarely been found in nature; **304** showed a neuroprotective effect through increasing the viability of A25-35-induced PC12 cells, whereas **305** exhibited selective inhibition against A549 with an IC_50_ value of 3.2 ± 0.1 μM [92]. A drimane sesquiterpenoid, astellolide Q (**308**) (Figure 7), was isolated from the mangrove-soil-derived *Penicillium* sp. N-5, combined with compound V [14]. A drimane sesquiterpene ester, chrysoride A (**309**) (Figure 7), was isolated from the red-alga-derived *P. chrysogenum* LD-201810 and showed moderate cytotoxicity against HepG2 and HeLa cancer cell lines, with IC_50_ values of 28.9 and 35.6 μM, respectively [93]. The marine-derived *Penicillium* sp. ZZ1283 yielded a drimane sesquiterpene, lactone purpuride D (**310**) (Figure 7), which significantly inhibited the growth of methicillin-resistant *S. aureus*, *E. coli* and *C. albicans* with MIC values of 4, 3 and 8 μg/mL, respectively [94]. Acorane-type sesquiterpenes feature a spiro[4.5]decane core with an isopropyl unit at C-1 and dimethyl substitution at C-4 and C-8, which markedly differs from other types of the sesquiterpene family [95]. Eighteen acorane-type sesquiterpenes, bilaiaeacorenols A–R (**311**–**328**) (Figure 7), were identified from the deep-sea-sediment-derived *P. bilaiae* F-28. Sesquiterpene **328** exhibited efficient reduction against NO production in LPS-induced BV-2 macrophages in a dose-dependent manner, and it abolished LPS-induced NF-*κ*B in the nucleus of BV-2 microglial cells, along with the inhibition of iNOS and COX-2 at a cellular level [95]. Citreobenzofurans D–F (**329**–**331**) and phomenones A–B (**332**–**333**) (Figure 7) were isolated from the mangrove-derived *Penicillium* sp. HDN13-494. Citreobenzofurans **330** and **331** are eremophilane-type sesquiterpenoids with rare benzofuran frameworks. Phomenone B (**332**) contained a rare thiomethyl group, which was the first report of this kind of sesquiterpene with sulfur elements in the skeleton; **333** showed moderate activity against *Bacillus subtilis*, with an MIC value of 6.25 μM [96]. (2*S*,3*S*,5*S*,6*S*,7*S*,8*R*,11*S*,12*R*)-15-deacetyl-7,8-dihydroxycalonectrin (**334**) and 1-methyl-4-[3,4,5-trihydroxy-1,2,2-trimethylcyclopently] benzene (**335**) (Figure 7) were isolated from the deep-sea-derived *Penicillium* sp. LXY140-R. 1-methyl-4-[3,4,5-trihydroxy-1,2,2-trimethylcyclopently] benzene (**335**) showed potent antiproliferative activity against HCT-116 cell lines with an IC_50_ value of 124.12 μM [97]. Nor-bisabolane derivative enantiomers (±)-1 (**336a**/**b**) (Figure 7) were isolated from the algal-derived *P. chrysogenum* LD-201810 [98]. Two drimane sesquiterpenes, (4*S*,5*R*,9*S*,10*R*)-11,13-dihydroxy-drim-7-en-6-one (**337**) and (4*S*, 5*R*,9*S*,10*R*)-11-hydroxy-13-carboxy-drim-7-en-6-one (**338**) (Figure 7), were isolated from the hydrothermal-vent-sediment-derived *Penicillium* sp. TW58-16. (4*S*,5*R*,9*S*,10*R*)-11,13-dihydroxy-drim-7-en-6-one (**337**) showed a strong *a*-glucosidase inhibitory effect with an inhibition rate of 35.4% [60].

#### 2.3.2. Diterpenes

Resistance has been found in many clinical anti-influenza A virus (IAV) drugs. Therefore, developing a safe and effective agent with a unique structure is urgently needed to combat IAV infection [99]. During the search for anti-IAV marine natural products, a series of new indole-diterpenoids have been isolated. Penijanthine E (**339**) (Figure 7), obtained from the marine-derived *P. citrinum* ZSS-9, showed antiviral activity against IAV of A/WSN/33(H1N1) and A/PR/8/34(H1N1) strains with IC_50_ values of 12.6 and 18.9 μM, respectively [99]. The marine-derived *P. janthinellium* co-cultured with *Paecilomyces formosus* led to the isolation of janthinellumines A–I (**340**–**348**). Janthinellumines **340**, **341** and **346** (Figure 7) displayed significant activities against two strains of A/WSN/33 (H1N1) and A/Hong Kong/1/68 (H3N2) with IC_50_ values of 3.8 and 13.3 μM, respectively, stronger than those of the positive control T-705. Furthermore, the PTP inhibitory activity of **340**–**341**, **343**–**344** and **348** had the best inhibitory activity towards PTP1B with IC_50_ values ranging from 0.6 to 9.2 μM, most of which were stronger than that of the positive control Na_3_VO_4_ (IC_50_ = 8.5 μM) [100]. Additionally, oxalierpenes A (**349**) and B (**350**) (Figure 7) were obtained from the mantis-shrimp-derived *P. oxalicum*. Oxalierpene A (**349**) represents the first indole-diterpenoid derivative with a five-membered ring of 4-hydroxy-5,5-dimethyl-dihydrofuran-3-one as a side chain. Oxalierpene B (**350**) had a unique 6/5/6/5/5/6/6/5/5 ring system. Oxalierpenes A (**349**) and B (**350**) showed antiviral activity against the H1N1 virus and respiratory syncytial virus (RSV), with IC_50_ values ranging from 2.8 to 9.4 μM [101]. 4-Hydroxyleptosphin C (**351**) and 13-*epi*-conidiogenone F (**352**) (Figure 7) were isolated from the marine-sediment-derived *P. antarcticum* KMM 4670. 4-Hydroxyleptosphin C (**351**) and 13-*epi*-conidiogenone F (**352**) inhibited *C. albicans* growth by 30.4% and 27.9% at 12.5 μM, respectively. Moreover, they significantly inhibited sortase A activity by 28.2% and 36.9% at 50 μM, respectively [102]. Shearinines R–T (**353**–**355**) and 22-hydroxyshearinine I (**356**) (Figure 7) were isolated from mangrove-sediment-derived *Penicillium* sp. UJNMF0740 [103]. Conidiogenones J (**357**) and K (**358**) (Figure 7) were isolated from the mangrove-derived *P. oxalicum* HLLG-13. Both of them showed significant growth inhibition activities against newly hatched *Helicoverpa armigera* Hubner larvae, with IC_50_ values of 200 μg/mL [15]. Penerpenes K–N (**359**–**362**) (Figure 7) were isolated from the bivalve mollusk-derived *Penicillium* sp. KFD28 [104]. Epipaxilline (**363**) and penerpene J (**364**) (Figure 7) were isolated from the marine-derived *Penicillium* sp. KFD28. Epipaxilline (**363**) and penerpene J (**364**) showed inhibitory activities against PTP1B with IC_50_ values of 31.5 and 9.5 μM, respectively. Penerpene J (**364**) also showed inhibitory activities against TCPTP with an IC_50_ value of 14.7 μM [105].

#### 2.3.3. Meroterpenes

**Figure 8:**
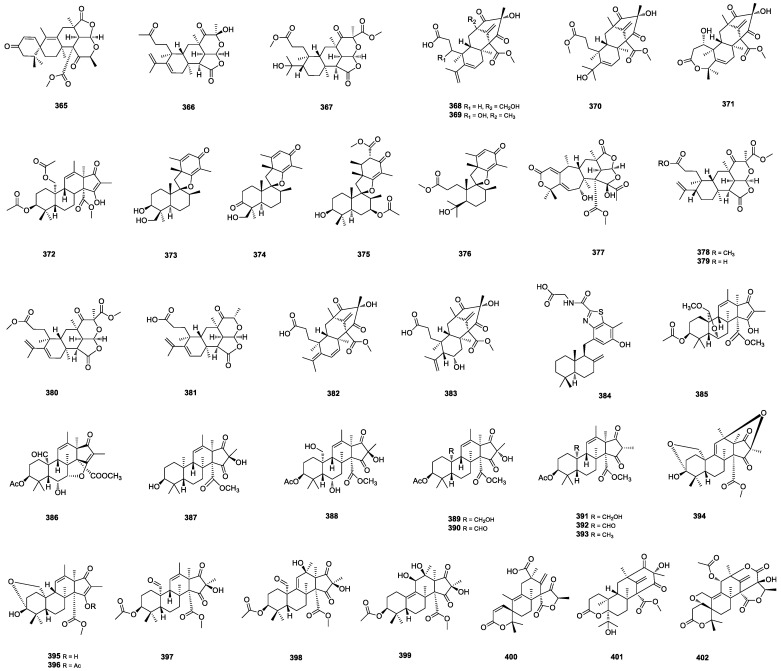
Structures of **365**–**402**.

Meroterpenoids are a family of hybrid natural products with high scaffold diversity and significant pharmacological activities [106]. Peniscmeroterpenoids H–N (**365**–**371**) (Figure 8) were isolated from the marine-derived *P. sclerotiorum* GZU-XW032. Among them, **365** featured a unique 2-oxaspiro[5.5] undeca-4,7-dien-3-one motif. Peniscmeroterpenoids **366** and **367** owned rare 6(D)/5(E) fused rings. Peniscmeroterpenoid **368** was the first case where the C-24 was oxidized. Peniscmeroterpenoid **369** exhibited a moderate inhibitory effect in NO production with an IC_50_ value of 48.04 ± 2.51 μM [106]. Andrastin I (**372**) (Figure 8) was isolated from the seafloor-sand-derived *P. ochrochloron* [107]. Chermesins E–H (**373**–**376**) (Figure 8) were isolated from the alga-derived *P. chermesinum* EN-480. Chermesin **373** showed effective activities against the aquatic pathogens *E. tarda* and *V. anguillarum* with MIC values of 0.5 μg/mL, respectively. Chermesin **374** showed powerful activities against human pathogenic bacterium *E. coli* with an MIC value of 1 μg/mL [108]. Seven meroterpenoids, peniscmeroterpenoids A–G (**377**–**383**) (Figure 8), were isolated from the marine-derived *P. sclerotiorum* GZU-XW03-2. Peniscmeroterpenoid **377** possessed an unprecedented and highly oxidized 6/7/6/5/5 pentacyclic system, featuring a unique tetrahydrofuro [2,3-b]furan-2(3H)-one motif. Peniscmeroterpenoids **378**–**381** with 6(D)/5(E) fused rings were rare in natural products, and **381** was the first example of a berkeleyone analogue stripped of the methyl ester fragment. In bioassays, **377** and **380** inhibited the production of NO in RAW264.7 cells with IC_50_ values of 26.60 ± 1.15 and 8.79 ± 1.22 μM. Moreover, **380** significantly suppressed the production of pro-inflammatory mediators (COX-2, IL-1*β* and IL-6) and the protein expression of the enzyme iNOS [109].

Meroterpenthiazole A (**384**) [110] and nine andrastones, namely citrehybridonol B (**385**), andrastin G (**386**), and andrastones B–H (**387**–**393**) [111] (Figure 8), were isolated from the deep-sea-derived *P. allii-sativi* MCCC 3A00580. Meroterpenthiazole A (**384**) had a rare benzothiazole moiety and significantly inhibited the transcriptional effect of retinoid X receptor (RXR)-*α* (KD = 12.3 μM). Citrehybridonol B (**385**) had a novel hemiketal moiety, and **386** was the first example to possess a novel tetrahydrofuran moiety via C-7 and C-15. Andrastones **391**–**393** were the first three examples of andrastones bearing a doublet methyl (C-18) at C-16; **387** potently decreased degranulation with an IC_50_ value of 40.4 μM. Three andrastin-type meroterpenoids, hemiacetalmeroterpenoids A–C (**394**–**396**) (Figure 8), were isolated from the mangrove-soil-derived *Penicillium* sp. N-5. Hemiacetalmeroterpenoid A (**394**) possessed a unique and highly congested 6,6,6,6,5,5-hexa-cyclic skeleton and exhibited significant antimicrobial activities against *P. italicum* and *C. gloeosporioides* with an MIC value of 6.25 μg/mL [14]. The other three andrastin-type meroterpenoids, penimeroterpenoids A–C (**397**–**399**) (Figure 8), were isolated from the deep-water-sediment-derived *Penicillium* sp. Penimeroterpenoid A (**397**) showed moderate cytotoxicity against the A549, HCT116, and SW480 cell lines [112]. The unusual austins-type meroterpenoids penicianstinoids C–E (**400**–**402**) (Figure 8) were obtained from the mangrove-derived *Penicillium* sp. TGM112. Penicianstinoid **400** owns two unusual spirocyclic moieties, **401** contains an octahydro-2*H*-chromen-2-one unit, and **402** has an uncommon five-membered ether ring. Penicianstinoids **400** and **402** inhibited the growth of newly hatched *H. armigera* Hubner larvae with IC_50_ values of 100 and 200 μg/mL, respectively [113].

### 2.4. Steroids

**Figure 9:**
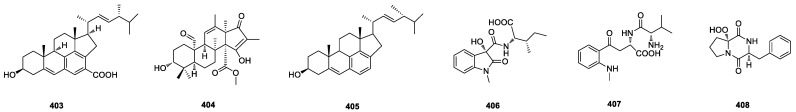
Structures of **403**–**408**.

Rubensteroid A (**403**) (Figure 9) was isolated from the Magellan Seamount-derived *P. rubens* AS-130. Rubensteroid A (**403**) had a rare 6/6/6/6/5 pentacyclic system and exhibited strong antibacterial activity against *E. coli* and *Vibrio parahaemolyticus*, both with an MIC value of 0.5 μg/mL [114]. Andrastin H (**404**) (Figure 9) was isolated from the mangrove-derived *P. oxalicum* HLLG-13 and showed significant growth inhibition activity against newly hatched *H. armigera* Hubner larvae, with an IC_50_ value of 50 μg/mL [15]. A unique 6/6/6/6/5 steroid, solitumergosterol A (**405**) (Figure 9), was isolated from the deep-sea-sediment-derived *P. solitum* MCCC 3A00215 [115].

### 2.5. Peptides

Two linear peptides, penicamides A (**406**) and B (**407**) (Figure 9), were isolated from the soft-coral-derived *Penicillium* sp. SCSIO 41512 [21]. Penicillizine A (**408**) (Figure 9) was isolated from the from the Red Sea tunicate-derived *P. commune* DY004 [116].

### 2.6. Others

**Figure 10:**
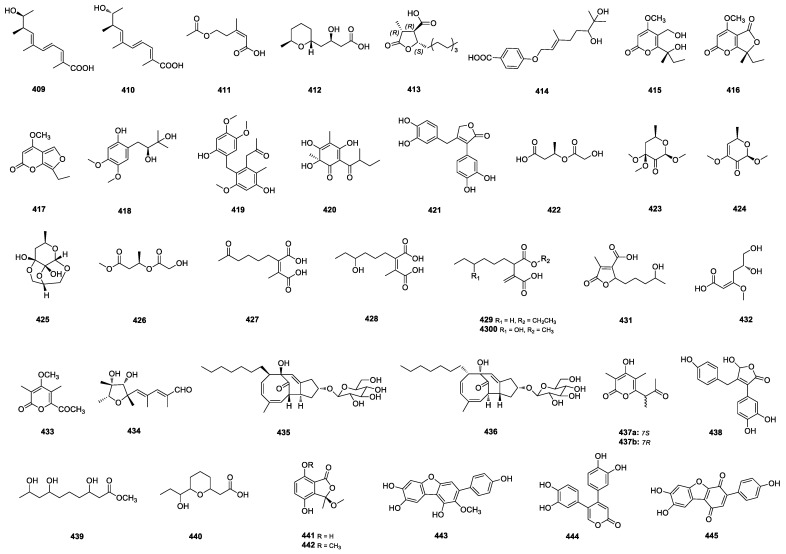
Structures of **409**–**445**.

Penioxa acids A (**409**) and B (**410**) (Figure 10) were isolated from the marine-sediment-derived *P. oxalicum* BTBU20213011 [117], and (*Z*)-5-acetoxy-3-methylpent-2-enoic acid (**411**) (Figure 10) was isolated from the mangrove-derived *P. oxalicum* HLLG-13 with a new natural product (2-hydroxy-5-methoxyphenyl) methyl acetate [15]. Antaketide A (**412**) (Figure 10) was isolated from the marine-sediment-derived *P. antarcticum* KMM 4670 [102]. A butyrolactone congener ochrochloronic acid (**413**) (Figure 10) was yielded from the seafloor-sand-derived *P. ochrochloron* co-cultivating with *Bacillus subtilis* [107]. (*Z*)-4-((6,7-dihydroxy-3,7-dimethyloct-2-en-1-yl)oxy)benzoic acid (**414**) (Figure 10) was isolated from a marine-mud-derived *P. arabicum* ZH3-9 [118]. Three *α*-pyrone derivatives, annularins L–N (**415**–**417**) (Figure 10), were isolated from the rhizospheric soil of the mangrove-derived *P. herquei* MA-370 [119]. Peniprenylphenol A (**418**) (Figure 10), a tetrasubstituted benzene derivative, was isolated from the mangrove-sediment-derived *P. chrysogenum* ZZ1151 and had antimicrobial activities against human pathogenic methicillin-resistant *S. aureus*, *E. coli* and *C.albicans* with MIC values of 6, 13, and 13 μg/mL, respectively [120]. 13-(11-Hydroxy-8-(4-hydroxy-1,6-dimethoxybenzyl)-9 -methoxy-12-methylphenyl) propan-15-one, a benzene derivate (**419**) (Figure 10), was isolated from the green-algae-derived *P. steckii* SCSIO 41040 [63]. A phloroglucinol derivative, speradine J (**420**) (Figure 10), was isolated from soft-coral-derived *Penicillium* sp. SCSIO 41038 [83]. A butenolide derivative, eutypoid F (**421**) (Figure 10), was isolated from the sponge-derived *Penicillium* sp. SCSIO 41413 and exhibited an inhibitory effect against the enzyme PI3K with an IC_50_ value of 1.7 μM [82]. Penisterines A (**422**) and C–E (**423**–**425**) and penisterine A methyl ether (**426**) (Figure 10) were isolated from the brown-alga-derived *P. sumatraense* SC29. Penisterine E (**425**) was a unique 6/6/6-tricyclic ether with an acetal and two hemiketal functionalities. Among these, **424** inhibited human endothelial progenitor cell (EPC) growth, migration, and tube formation without any cytotoxic effect. Furthermore, in in vivo bioassays, the percentages of angiogenesis of **423** on *Tg*(*fli1:EGFP*) transgenic zebrafish were 54% and 37% as the treated concentration increased from 10.2 to 20.4 μg/mL, respectively. The percentages of angiogenesis of **424** were 52% and 41% as the treated concentration increased from 8.6 to 17.2 μg/mL, respectively [121]. Five alkane derivatives (**427**–**431**) (Figure 10) were isolated from the mangrove-sediment-derived *P. ludwigii* SCSIO 41408. Alkane derivatives **429** and **431** exhibited obvious inhibitory activities against LPS-induced NF-*k*B with IC_50_ values of 10.7 and 21.5 μM, respectively. Moreover, in a further study of their effects on RANKL-induced osteoclastogenesis, alkane derivative **429** was found to be able to suppress the RANKL-induced osteoclast differentiation in BMMCs, with a concentration of 10 μM [80].

5,6-dihydroxy-3-methoxyhex-2-enoic acid (**432**) (Figure 10) was isolated from the deep-sea-sediment-derived *Penicillium* sp. LXY140-3 co-culturing with *Penicillium* sp. LXY140-R [97]. 6-acetyl-4-methoxy-3,5-dimethyl-2H-pyran-2-one (**433**) and (2*E*,4*E*)-5-((2*S*, 3*S*,4*R*,5*R*)-3,4-dihydroxy-2,4,5-trimethyltetrahydrofuran-2-yl)-2,4-dimethylpenta-2,4-dienal (**434**) (Figure 10) were identified from the mangrove-derived *P. polonicum* H175, and they showed no hypoglycemic effect by the *Tg (Ins: htBidTE-ON; LR)* zebrafish [122]. 5-glycopenostatins F (**435**) and I (**436**) (Figure 10), characterized by an unprecedented PKS scaffold bearing a glucose unit, were isolated from the sponge-derived *P. Copticola*. Their activities have not been identified [92]. (±)-Tetraketides **437a**/**b** were isolated from the sponge-derived *Penicillium* sp. SCSIO 41302. (-)-Tetraketide **437b** exhibited significant inhibitory activities against pancreatic lipase and acetyl cholinesterase with an IC_50_ value of 48.5 μM, which indicated that different chiral centers between enantiomers (**437a**/**b**) may result in different biological activities (IC_50_ value of **437** against PL > 100 μg/mL) [36]. 8-hydroxyhelvafuranone (**438**), methyl-3,7,9-trihydroxydecanate (**439**), and 9-hydroxy-3,7-epoxydecanoic acid (**440**) (Figure 10) were isolated from the marine-sediment-derived *P. griseofulvum* MCCC 3A00225 [42]. Phthalides chrysoalides A (**441**) and B (**442**) (Figure 10) were isolated from the red-alga-derived *P. chrysogenum* LD-201810 [98]. *P*-terphenyl derivatives peniterphenyls A–C (**443**–**445**) (Figure 10) were obtained from the deep-sea-sediment-derived *Penicillium* sp. SCSIO 41030. Peniterphenyl **444** represents the first reported natural product possessing a 4,5-diphenyl-substituted 2-pyrone derivative. Peniterphenyls **443** and **444** significantly increased the viability of Vero cells infected with HSV-1/2 with the EC_50_ values of the *p*-terphenyls ranging from 1.4 ± 0.6 to 9.3 ± 3.7 μM, with 50% cytotoxicity concentration values greater than 100 μM against Vero cells [123].

The names and numbers of all new compounds according to their classes, the sources from which marine-derived *Penicillium* were isolated, the biological activities of new compounds, and corresponding references are listed in Table 1.

## 3. Statistical Analysis of New Natural Products from Marine-Derived *Penicillium*

**Figure 11:**
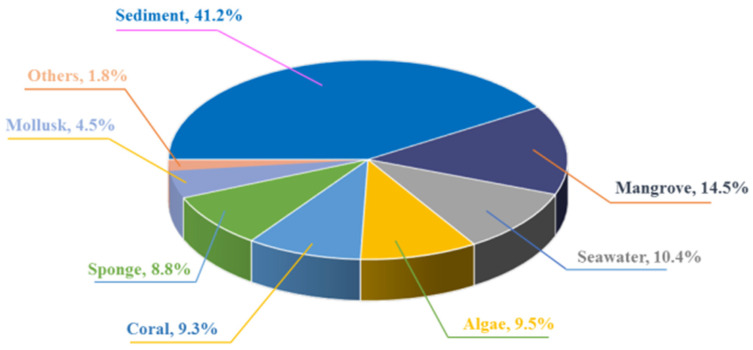
The proportion of the *Penicillium* fungi derived from different marine habitats.

*Penicillium* fungi can establish a good relationship with different marine organisms and marine environments. According to the statistical results, sediments and mangroves were the main sources or hosts of marine-derived *Penicillium* fungi for producing new natural products, nearly 56% (Figure 11).

**Figure 12:**
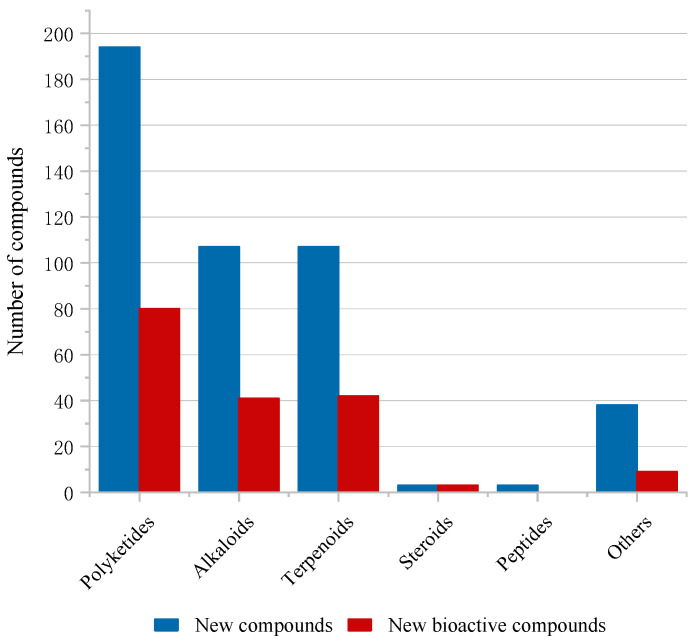
The proportion of new bioactive compounds in each chemical class.

The new natural products had diverse chemical structures including polyketides, alkaloids, terpenoids, steroids, peptides, and others. Figure 12 shows the proportion of new bioactive compounds in each chemical class. A total of 194, 107 and 107 new compounds belong to polyketides, alkaloids and terpenoids, respectively, adding up to more than 90% of the total. Similarly, these three classes contribute 94% of all new bioactive compounds. The highest proportion of bioactives belongs to the largest number of polyketides (86) with 44.3%, followed by terpenoids (41) with 39.3% and alkaloids (42) with 38.3%.

The new compounds were counted only once when they were analyzed for bioactivity or inactivity. If the article did not provide a description of strong, moderate or weak activity of bioactive compounds, we gave this description according to bioactivity potency criteria used in the review [124]. The multi-active compounds were counted multiple times when they were classified according to anti-cancer/cytotoxicity, anti-inflammatory, antibacterial, antifungal, antiviral, enzyme inhibitory, antioxidant, and anti-allergy activities, as well as others [125].

**Figure 13:**
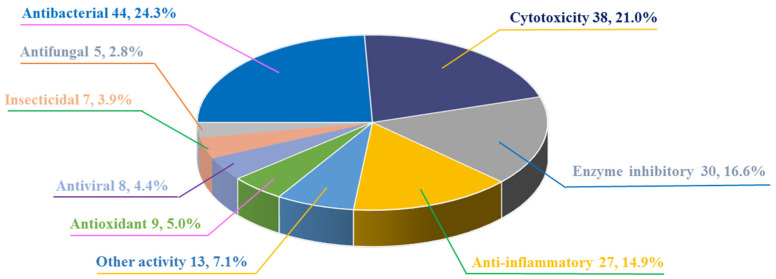
The percentage distribution of new compounds with different bioactivities.

Figure 13 shows the percentage distribution of new compounds with different bioactivities for 2021–2023. Among them, 24.3% of the new bioactive compounds showed antibacterial activity with the number of 44. This was followed by cytotoxic activity at 38 (21%), enzyme inhibition activity at 30 (16.6%), and anti-inflammatory activity at 27 (14.9%).

**Figure 14:**
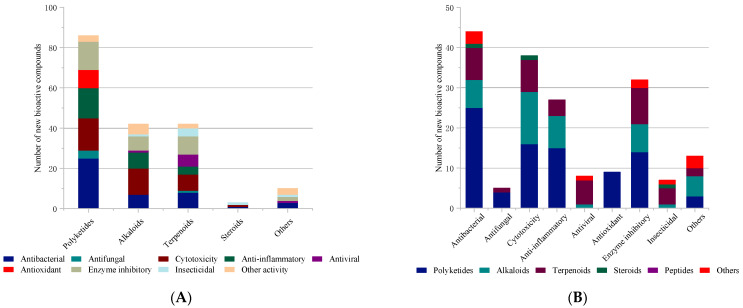
(**A**) The proportion distribution of new compounds with different bioactivities in each chemical class; (**B**) the proportion distribution of new compounds with different chemical classes in each bioactivity.

Figure 14A shows the proportion distribution of new compounds with different bioactivities in each chemical class for 2021–2023; peptides are not listed due to the absence of activity results. Polyketides displayed antibacterial activity as the dominant activity with a proportion of 29%, highlighting that they encompass many potential antibacterial drug leads. For alkaloids, cytotoxic compounds accounted for 31.7% of the total active compounds, while terpenoids displayed relatively high enzyme inhibitory with a proportion of 21.4%. Figure 14B shows the proportion distribution of new compounds with different chemical classes in each bioactivity for 2021–2023. The major contributors to antibacterial activity are polyketides. The most promising anti-cancer/cytotoxicity agents from marine-derived *Penicillium* fungi appear to be alkaloids. The main anti-inflammatory and enzyme inhibitory metabolites are still polyketides.

It should be noted that not all new metabolites isolated from the marine-derived *Penicillium* fungi were tested for biological activity because of scarcity of quantity [37,52,59,79,92], while many bioactive compounds were only studied for one type of bioassay. In addition, most of the biological activities of the experimental subjects are performed in vitro. Correspondingly, bioactive assays in vivo are only applied in a few studies, for example, zebrafish models used for investigations into anti-cancer/cytotoxicity [66], anti-angiogenesis [86,121], and hypoglycemia [122]. Furthermore, the difficulty of the biological screening model was another factor affecting the screening result. In fact, viruses were not considered as screening targets in general laboratory due to inherent complexity of cell-based assays of viruses [125], while mice models were expensive and time consuming. Therefore, more new natural products from the marine-derived *Penicillium* fungi should be screened on a wider variety of bioassays, as effective enrichment of trace compounds and enhanced methods in bioactivity screening technologies are important.

## 4. Conclusions

This article provided a comprehensive overview of the source, chemistry, and bioactivities of 452 secondary metabolites from marine-derived *Penicillium* fungi described from 2021 to 2023. Although the coronavirus disease 2019 (COVID-19) pandemic limited opportunities for field collections in domestic and international travel, the numbers of new compounds from marine-derived *Penicillium* fungi increased abundantly, compared to 578 new compounds reported from 1991 to 2020 [6,7]. This trend might be associated with fungal large-scale cultures under laboratory conditions and the significant impact of penicillin, the first broad-spectrum antibiotic in drug development [126]. In addition, fungal culture methods, extraction and separation techniques, structure identification technology, and biological screening methods have reached a relatively mature level [125].

New methods and in-depth research on important compounds have been carried out. Affected by the COVID-19 pandemic, both pathways of TNF-*α*-induced NF*κ*B activation and TGF-*β*-induced Smad activation were applied to evaluate azaphilone compounds for the first time [23]. The HPLC-MS/MS analyses [9], one-pot/two-stage precursor-directed biosynthesis approach [88], and molecular networks of MS/MS data generated with Global Natural Products Social Molecular Networking (GNPS) [13] have expanded the scope of research on metabolites, especially trace components of marine-derived *Penicillium* fungi. Co-culture [66,97,100,107] and OSMAC [17,18,20,111] have been used to explore the structural diversity of secondary metabolites from the fungi. Further research on the known compounds, whether penicopeptide A as a candidate compound for osteoporosis prevention [12] or the anti-pancreatic cancer activity of dicitrinone G evaluated using a mouse model [11], provides an opportunity to diversify the targets, increasing the value of natural products from marine-derived *Penicillium* fungi.

In summary, marine-derived *Penicillium* fungi resources are found worldwide and have attracted great attention due to their diverse chemical structures. *Penicillium* fungi have produced a large number of structurally novel and bioactively potent compounds, such as polyketides, alkaloids and terpenoids. Over a thousand secondary metabolites from marine-derived *Penicillium* fungi have already been reported in the past thirty-three years (1991–2023). Although none of them have reached the market yet, which could partly be related to non-comprehensive screening approaches and a lack of sustained lead optimization, the mass production of trace amounts of compounds by symbiotic *Penicillium* fungi and the symbiotic relationship with the marine host make marine-derived *Penicillium* fungi a very important source of bioactive compounds for drug discovery.

## Figures and Tables

**Table 1:** New compounds from marine-derived *Penicillium* fungi.

No.	Compounds	Fungal Species/Strain No.	Source of Fungi	Bioactivities	Ref.
**Azaphilones**					
**1**	Penicilazaphilone Ia	*P. sclerotiorum* E23Y-1A	Sponge	Cytotoxic activity Anti-inflammatory	[17]
**2**	Penicilazaphilone J				
**3**	*epi*-geumsanol D				
**4**–**5**	Penidioxolanes C–D				
**6**–**9**	Penicilazaphilone K–N				
**10**–**11**	Penicilazaphilones F–G	*P. sclerotiorum* E23Y-1A	Sponge	Anti-inflammatory	[22]
**12**	5-bromoisorotiorin	*P. sclerotiorum* E23Y-1A	Sponge	Antibacterial activity Enzyme inhibitory	[18]
**13**	Penicilazaphilone Ha				
**14**	Penicilazaphilone Hb	*P. sclerotiorum*	Algae	Anti-angiogenesis	[20]
**13**	Penicilazaphilone Ib				
**16**	11-*epi*-geumsanol F				
**17**	11-*epi*-geumsanol B				
**18**	8a-*epi*-hypocrellone A	*P. sclerotiorum*	Sediment	Cytotoxic activity Anti-inflammatory	[23]
**19**	8a-*epi*-eupenicilazaphilone C				
**20a**/**b**	Isochromophilone H (a/b) (isomers)	*P. sclerotiorum* HY5	Mangrove	Phytotoxicity	[24]
**21**	Sclerotiorin A				
**22**	Sclerotiorin B				
**23**	Ochlephilone				
**24**	Isochromophilone IV				
**25a**/**b**	Isochromophilone J (a/b) (isomers)				
**26**–**29**	Chermesinones D–G	*P. chermesinum* FS625	Seawater	Anti-inflammatory	[25]
**30**–**31**	Daldinins G–H	*P. glabrum* glmu 003	Soft coral	Antibacterial activity Enzyme inhibitory	[26]
**Isocoumarins**					
**32**–**33**	Peniciisocoumarins I–J	*Penicillium* sp. GXIMD 03001	Mangrove	Cytotoxic activity	[27]
**34**–**35**	Penicillols A–B	*Penicillium* sp. BJR-P2	Mangrove	Anti-inflammatory	[28]
**Chromones**					
**36**–**38**	Epiremisporines C–E	*P. citrinum* BCRC 09F458	Waste water	Cytotoxic activity Anti-inflammatory	[29]
**39**–**41**	Epiremisporines F–H	*P. citrinum* BCRC 09F458	Waste water	Cytotoxic activity Anti-inflammatory	[30]
**42**–**49**	Penithochromones M–T	*P. thomii* Maire YPGA3	Sediment	Enzyme inhibitory Antioxidant activity	[31]
**50**–**52**	Penithochromones U–W	*P. thomii* YPGA3	Sediment	Enzyme inhibitory	[32]
**Citrinins**					
**53**	(5*R*)-and (5*S*)-isoquinocitrinin B	*Penicillium* sp. TW131-64	Sediment	Antibacterial activity	[33]
**54**	(5*R*)-and (5*S*)-isoquinocitrinin C				
**55**	(5*R*)-and (5*S*)-isoquinocitrinin D				
**56**	(3*R*,4*S*)-8-hydroxy-6-methoxy-3,4,5-trimethylisochromane-7-carboxylatemethyl				
**57**	(3*R*,4*S*)-6-hydroxy-8-methoxy-3,4,5-trimethylisochromane-7-carboxylatemethyl				
**58**	Penicitrinone J				
**59**–**62**	Dicitrinone G–J	*Penicillium* sp. GGF16-1-2	Starfish	Antifungal and cytotoxic activities	[10]
**63**–**65**	Neotricitrinols A–C	*P. citrinum* W23	Sediment	Anti-osteoporosis activity	[34]
**66**–**67**	Xerucitrinins B–C	*P. citrinum* Y34	Sediment	Enzyme inhibitory	[35]
**68**	Penicitrinol P	*Penicillium* sp. SCSIO 41302	Sponge	Antibacterial activity Enzyme inhibitory	[36]
**69**	Dicitrinol D	*Penicillium* sp. SCSIO 41303	Sponge	Antibacterial, cytotoxic, antiviral activities, and enzyme inhibitory	[37]
***β*-resorcylic acids**					
**70**	14-hydroxyasperentin B	*P. antarcticum* KMM 4685	Brown alga	Cytotoxic activity	[38]
**71**–**73**	*β*-resoantarctines A–C				
**74**	8-dehydro-*β*-resoantarctine A				
**Verrucosidins**					
**75**–**76**	9-*O*-ethylpenicyrones A–B	*P. cyclopium* SD-413	Sediment	Antibacterial activity	[40]
**77**–**82**	Poloncosidins A–F	*P. polonicum* CS-252	Sediment	Antibacterial activity	[39]
**83**–**87**	Poloncosidins G–K	*P. polonicum* CS-252	Sediment	Antibacterial activity	[41]
**88**–**89**	Verrucosidinol A–B	*P. griseofulvum* MCCC 3A00225	Sediment	Anti-food allergy	[42]
**Citreoviridins**					
**90**–**91**	Citreoviridins H–I	*Penicillium* sp. BJR-P2	Mangrove	Anti-inflammatory	[27]
**92**–**97**	Citreoviridins J–O	*P. citreonigrum* MCCC 3A00169	Sediment	Cytotoxic activity Anti-inflammatory	[43]
**Nitrogen-containing polyketides**					
**98**–**99**	Oxopyrrolidines A–B	*P. oxalicum*MEFC104	Sediment	Antibacterial activity	[44]
**100**	7-hydroxy-3,10-dehydrocyclopeptine	*P. polonicum* MCCC3A00951	Sediment	Antiviral activity	[45]
**101**–**105**	Steckfusarins A–E	*P. steckii* SCSIO41040	Green algae	Antibacterial, antifungal, cytotoxic and antiviral activities Enzyme inhibitory Antioxidant Anti-inflammatory	[46]
**Sorbicillinoids**					
**106**–**108**	Bisorbicillchaetones A–C	*Penicillium* sp. SCSIO06868	Sediment	Anti-inflammatory	[47]
**109**	10-Methylsorbiterrin A	*Penicillium* sp. SCSIO06871	Sediment	Antibacterial and antifungal activities Enzyme inhibitory	[48]
**110**	Epitetrahydrotrichodimer ether				
**111**	Demethyldihydrotrichodimerol				
**112**	Bisorbicillpyrone A				
**113**	Dihydrotrichodermolidic acid				
**114**	5-hydroxy-dihydrodemethy lsorbicillin				
**115**	Sorbicillpyrone A				
**116**	5,6-dihydrovertinolide				
**117**–**118**	Sorbicatechols C–D	*P. allii-sativi* MCCC3A00580	Seawater	Cytotoxic activity	[49]
**119**	(4*E*)-1-(4,6-Dihydroxy-5-methylpyridin-3-yl)hex-4-en-1-one	*Penicillium* sp. DM815	Mangrove	Anti-inflammatory	[50]
**Isochromans**					
**120**–**125**	Penicisteckins A–F	*P. steckii* HNNU-5B18	Beach mud	Antibacterial activity Cytotoxic activity	[51]
***α*-pyrone polyketides**					
**126**–**130**	Penipyrols C-G	*Penicillium* sp. HDN-11-131	Mangrove	Cytotoxic activity	[52]
**131**	Methyl-penipyrol A				
**Hirsutellones**					
**132**	Perpyrrospirone A	*P. citrinum*	Seawater	Cytotoxic activity	[53]
**Xanthones and benzophenones**					
**133** **134**	11-*O*-acetylaspergillusone B	*Penicillium* sp. MCCC 3A00126	Sediment	Cytotoxic activity Ferroptosis inhibitory	[54]
	7-dehydroxyhuperxanthone A				
**135**	Penicixanthene E	*Penicillium* sp. GXIMD 03101	Mangrove	Cytotoxic activity	[55]
**136**	Penibenzophenone C	*Penicillium* sp.	Mangrove	Antibacterial and insecticidal activities	[16]
**Hydroxybenzenes**					
**137**	Peniketide A	*Penicillium* sp. SCZ-1	Sediment	Enzyme inhibition	[56]
**138**	Methyl ester of penipyrol A				
**139**–**141**	Penidihydrocitrinins A–C	*P. citrinum* W17	Sediment	Anti-inflammatory Anti-osteoporosis	[57]
**142**	Peniciphenalenin G	*P. oxalicum*	Seawater	Cytotoxic activity	[58]
**143**	Penicinone C	*Penicillium* sp. LA032	Mangrove	----	[59]
**144**	5-((*R*,1*Z*,3*E*)-6-hydroxy-1,3-heptadien-1-yl)-1,3-benzenediol	*Penicillium* sp. TW58-16	Sediment	Anti-inflammatory Enzyme inhibition	[60]
**145**	4-carboxy-5-((*R*,1*Z*,3*E*)-6-hydroxy-1,3-heptadien-1-yl)-1,3-benzenediol				
**146**	4-carboxy-5-((1*Z*,3*E*)-1,3-heptadien-1-yl)-1,3-benzenediol				
**147**	5-((1*Z*,3*E*)-4-carboxy-1,3-butadienyl-1-yl)-1,3-benzenediol				
**148**	(2*E*)-3-[(3*R*)-3,4-dihydro-6,8-dihydroxy-1-oxo-1H-2-benzopyran-3-yl]-2-propenoic acid				
**149**	3-[(3*S*)-3,4-dihydro-6,8-dihydroxy-1-oxo-1H-2-benzopyran-3-yl]-propanoic acid				
**150**	Coniochaetone N	*Penicillium* sp. SCSIO06868	Sediment	Antibacterial activity	[61]
**Lactones**					
**151**–**152**	Penicinones A–B	*Penicillium* sp. LA032	Mangrove	Cytotoxic activity	[59]
**153**	Walterolactone E	*Penicillium* sp. TW58-16	Sediment	Antibacterial activity	[62]
**Olefinic acids and their derivatives**					
**154**–**157**	Steckwaic acid A-D	*P. steckii* AS-324	Coral	Antibacterial and antifungal activities	[64]
**158**	11-ketotanzawaic acid D				
**159**	6,15-dihydroxytanzawaic acid M				
**160**	15*R*-methoxytanzawaic acid M				
**161**	15*S*-methoxytanzawaic acid M				
**162**	8-hydroxytanzawaic acid M				
**163**	8-hydroxytanzawaic acid B				
**164**–**168**	Steckwaic acid Ea–Ia	*P. steckii* AS-324	Coral	Antibacterial activity	[19]
**169**	18-O-acetyltanzawaic acid R				
**170**	10-hydroxytanzawaic acid U				
**171**	13*R*-tanzawaic acid S				
**172**–**176**	Steckwaic acid Eb–Ib	*P. steckii* SCSIO 41040	Green algae	Antibacterial, antifungal, cytotoxic, and antiviral activities	[63]
**177**–**178**	Steckwaic acid J–K				
**179**–**182**	Penicisteck acid A–D	*P. steckii* SCSIO 41025	Mangrove	Antibacterial activity Enzyme inhibition	[65]
**183**–**186**	Penifellutins A–D	*P. crustosum* PRB-2 and *P. fellutanum* HDN14-323	Seawater	Cytotoxic activity	[66]
**Other polyketides**					
**187**	Rubenpolyketone A	*P. rubens* AS-130	Coral	Antibacterial activity	[67]
**188**	Oxalichroman A	*P. oxalicum* 2021CDF-3	Red algae	Cytotoxic activity	[68]
**189**	Oxalihexane A				
**190**	Leptosphaerone D	*Penicillium* sp. TW58-16	Sediment	Antibacterial activity	[62]
**191**	15-*O*-methyl ML-236A	*P. solitum* MCCC 3A00215	Sediment	Cytotoxic activity Anti-food allergy	[69]
**Indole alkaloids**					
**192**–**195**	Communesins M–P	*P. expansum* MMS42	Sediment	Cytotoxic and neuroprotective activities	[9]
**196**	Deoxy-14,15-dehydroisoaustamide	*P. dimorphosporum* KMM 4689	Soft coral	Cytotoxic activity	[70]
**197**	16*α*-hydroxy-17*β*-methoxy-deoxydihydroisoaustamide	*P. dimorphosporum* KMM 4689	Soft coral	Cytotoxic and neuroprotective activities	[71]
**198**	16*β*-hydroxy-17*α*-methoxy-deoxydihydroisoaustamide				
**199**	16*β*,17*α*-dihydroxy-deoxydihydroisoaustamide				
**200**	16*α*-hydroxy-17*α*-methoxy-deoxydihydroisoaustamide				
**201**	16*α*,17*α*-dihydroxy-deoxydihydroisoaustamide				
**202**	16,17-dihydroxydeoxydihydroisoaustamide				
**203**	3*β*-hydroxy-deoxyisoaustamide				
**204**	Penilline D	*Penicillium* sp. SCSIO 05705	Soil	Antibacterial and cytotoxic activities Enzyme inhibition	[72]
**205**	Penindolacid A	*Penicillium* sp. LW92	Sediment	Antioxidant activity Enzyme inhibitory	[73]
**206**–**207**	Penicamides A–B	*Penicillium* sp. LA032	Soil	Cytotoxic activity	[59]
**208**	11*S*-(−)-penilloid A	*Penicillium* sp. ZZ1750	Marine mud	Cytotoxic activity	[74]
**209**	11*R*,14*E*-(+)-penilloid A				
**Pyridones**					
**210**–**220**	Penicipyridones A–K	*P. oxalicum* QDU1	Leaves of plant	Anti-inflammatory	[75]
**Quinolinones**					
**221**	Penicinolone	*Penicillium* sp. SCSIO 41033	Sponge	Antibacterial and antifungal activities Enzyme inhibitory	[76]
**222**	Quinolactone A	*P. citrinum* YX-002	Mangrove	Enzyme inhibitory	[77]
**223**	Quinolactacin C1				
**224**	3-*epi*-quinolactacin C1				
**225a**/**b**	Quinolactacin E (a racemic mixture)	*Penicillium* sp. SCSIO 41303	Sponge	Cytotoxic and antiviral activities Enzyme inhibitory	[37]
**226**	Quinolactacin F1				
**227**	Quinolactacin F2				
**228a**/**b**	Quinolactacin G (enantiomers)				
**229**–**232**	(±)-oxypenicinolines A–D	*P. steckii* SCSIO 41025	Mangrove	Antibacterial, antifungal, and cytotoxic activities Enzyme inhibitory	[78]
	(racemic mixtures, respectively)				
**233**–**234**	Penicinoline F–G				
**Decahydrofluorene-class alkaloids**					
**235**–**241**	Pyrrospirone K–Q	*Penicillium* sp. SCSIO 41512	Soft coral	Antibacterial and cytotoxic activities Enzyme inhibitory	[79]
**Piperazines**					
**242**	Adametizine C	*P. ludwigii* SCSIO 41408	Sediment	Antibacterial, antifungal, and cytotoxic activities Anti-osteoporosis	[80]
**243**	(8*S*,9*R*,12*R*,18*S*)-12-hydroxy-fumitremorgin B	*Penicillium* sp. TW58-16	Sediment	Antibacterial activity	[62]
**244**–**246**	Penigainamides A–C	*P. steckii* YE	Seawater	Cytotoxic activity	[81]
**Tetramic-acid-based alkaloids**					
**247**–**249**	Tolypocladenols D–F	*P. oxalicum* QDU1	Leaves of plant	Antifungal and cytotoxic activities Anti-inflammatory	[75]
**250**–**251**	Penicillenols G1–G2	*Penicillium* sp. SCSIO06868	Sediment	Antibacterial and antiviral activities	[61]
**252**	Penicillenol H				
**Amines and amides**					
**253**	(*Z*)-4-(5-acetoxy-N-hydroxy-3-methylpent-2-enamido) butanoate	*P. oxalicum* HLLG-13	Mangrove	Antibacterial and insecticidal activities	[15]
**254**–**255**	Polonimides D–E	*Penicillium* sp. SCSIO 41413	Sponge	Antibacterial and cytotoxic activities Anti-inflammatory	[82]
**256**	Speradine I	*Penicillium* sp. SCSIO 41038	Soft coral	Cytotoxic activity Enzyme inhibitory	[83]
**257**	(*S*)-2-acetamido-4-(2-(methylamino)phenyl)-4-oxobutanoic acid	*P. citrinum* XIA-16	Shrimp	Ferroptosis inhibitory	[84]
**258**	Citrinadin C	*P. citrinum*	Sediment	Antibacterial and cytotoxic activities	[85]
**259**	(2*S*,2′*R*,3*R*,3′*E*,4*E*,8*E*)-N-2′-hydroxyhexadecanoyl-2-amino-9-methyl-4,8-octadecadiene-1,3-diol	*P. chrysogenum* Y20-2	Seawater	Anti-angiogenesis	[86]
**260**–**261**	Penichryfurans A–B	*P. chrysogenum*	Red alga	Cytotoxic activity	[87]
**262**–**280**	Talaroenamines F1−F19	*P. malacosphaerulum* HPU-J01	Wetland	Cytotoxic activity	[88].
**281**	Peniokaramine	*Penicillium* sp. LSH-3-1	Sediment	Cytotoxic activity Anti-inflammatory	[89]
**282**	Penipyranopyridine				
**283**–**284**	Penicidihydropyridones A–B	*Penicillium* sp. B9	Sponge	Cytotoxic activity	[90]
**285**	(+)-solitumidine D	*P. solitum* MCCC 3A00215	Sediment	Cytotoxic activity Anti-food allergy	[69]
**286**	(±)-solitumidine E (a racemic mixture)				
**287**	Penicmariae-crucis C acid	*P. steckii* SCSIO 41025	Mangrove	Antibacterial and antifungal activities Enzyme inhibitory	[65]
**288**	*N*-(6-hydroxy-2-oxoindolin-3-ylidene)-5′-methoxy-5′-oxobutyl-amine oxide				
**289**	Methyl-1′-(*N*-hydroxyacetamido)-butanoate				
**290**	Penigrisamide	*P. griseofulvum* MCCC 3A00225	Sediment	Anti-food allergy	[42]
**291**	Aurantiomoate C				
**292**	*N*,*N*-pyroglutamylleucinmethylester				
**293**	Methyl 2S-hydroxy-3-methylbutanoyl-L-leucinate				
**294**	6*R*,7-dihydroxy-3,7-dimethyloctanamide				
**Other alkaloids**					
**295**	Sulfoxanthocillin	*Penicillium* sp. SCSIO sof101	Seawater	Antibacterial activity Anti-inflammatory	[91]
**296**	Penipyridinone B	*Penicillium* sp. ZZ1750	Sea mud	Cytotoxic activity	[74]
**Sesquiterpenes**					
**297**	Chermesiterpenoid D	*P. rubens* AS-130	Coral	Antibacterial activity	[67]
**298**–**307**	Copteremophilanes A–J	*P. Copticola*	Sponge	Cytotoxic activity Neuroprotection	[92]
**308**	Astellolide Q	*Penicillium* sp. N-5	Soil	Antibacterial and antifungal activities	[14]
**309**	Chrysoride A	*P. chrysogenum* LD-201810	Red alga	Cytotoxic activity	[93]
**310**	Purpuride D	*Penicillium* sp. ZZ1283	Sea mud	Antibacterial activity	[94]
**311**–**328**	Bilaiaeacorenols A–R	*P. bilaiae* F-28	Sediment	Anti-inflammatory	[95]
**329**–**331**	Citreobenzofurans D–F	*Penicillium* sp. HDN13-494	Soil	Antibacterial and cytotoxic activities	[96]
**332**–**333**	Phomenones A–B				
**334**	(2*S*,3*S*,5*S*,6*S*,7*S*,8*R*,11*S*,12*R*)-15-deacetyl-7,8-dihydroxycalonectrin	*Penicillium* sp. LXY140-R and *Penicillium* sp. LXY140-3	Sediment	Cytotoxic activity	[97]
**335**	1-Methyl-4-[3,4,5-trihydroxy-1,2,2-trimethylcyclopently]benzene				
**336a**/**b**	(±)Methylsulfinyl-1-hydroxyboivinianin A (enantiomers)	*P. chrysogenum* LD-201810	Red alga	Antifungal and cytotoxic activities	[98]
**337**	(4*S*,5*R*,9*S*,10*R*)-11,13-dihydroxy-drim-7-en-6-one	*Penicillium* sp. TW58-16	Sediment	Anti-inflammatory Enzyme inhibition	[60]
**338**	(4*S*,5*R*,9*S*,10*R*)-11-hydroxy-13-carboxy-drim-7-en-6-one				
**Diterpenes**					
**339**	Penijanthine E	*P. citrinum* ZSS-9	Sediment	Antiviral activity	[99]
**340**–**348**	Janthinellumines A–I	*P. janthinellum*	Seawater	Antibacterial and antiviral activity Enzyme inhibition	[100]
**349**–**350**	Oxalierpenes A–B	*P. oxalicum*	Shrimp	Antiviral activity	[101]
**351**	4-hydroxyleptosphin C	*P. antarcticum* KMM 4670	Sediment	Antibacterial activity Enzyme inhibition	[102]
**352**	13-*epi*-Conidiogenone F				
**353**–**355**	Shearinines R–T	*Penicillium* sp. UJNMF0740	Sediment	Antibacterial activity Neuroprotection	[103]
**356**	22-hydroxyshearinine I				
**357**–**358**	Conidiogenones J–K	*P. oxalicum* HLLG-13	Mangrove	Antibacterial and insecticidal activities	[15]
**359**–**362**	Penerpenes K–N	*Penicillium* sp. KFD28	Mollusk	Antibacterial and cytotoxic activities	[104]
**363**	Epipaxilline	*Penicillium* sp. KFD28	Mollusk	Enzyme inhibition	[105]
**364**	Penerpene J				
**Meroterpenes**					
**365**–**371**	Peniscmeroterpenoids H–N	*P. sclerotiorum* GZU-XW03-2	Mollusk	Anti-inflammatory	[106]
**372**	Andrastin I	*P. ochrochloron*	Seawater	Antibacterial activity	[107]
**373**–**376**	Chermesin E–H	*P. chermesinum* EN-480	Red alga	Antibacterial activity	[108]
**377**–**383**	Peniscmeroterpenoid A–G	*P. sclerotiorum* GZU-XW03-2	Mollusk	Anti-inflammatory	[109]
**384**	Meroterpenthiazole A	*P. allii-sativi* MCCC 3A00580	Seawater	Cytotoxic activity	[110]
**385**	Citrehybridonol B	*P. allii-sativi* MCCC 3A00580	Seawater	Anti-allergic bioactivity	[111]
**386**	Andrastin G				
**387**–**393**	Andrastones B–H				
**394**–**396**	Hemiacetalmeroterpenoids A–C	*Penicillium* sp. N-5	Soil	Antifungal activity	[14]
**397**–**399**	Penimeroterpenoids A–C	*Penicillium* sp.	Sediment	Cytotoxic activity	[112]
**400**–**402**	Penicianstinoids C–E	*Penicillium* sp. TGM112	Mangrove	Antifungal and insecticidal activities	[113]
**Steroids**					
**403**	Rubensteroid A	*P. rubens* AS-130	Coral	Antibacterial activity	[114]
**404**	Andrastin H	*P. oxalicum* HLLG-13	Mangrove	Insecticidal activity	[15]
**405**	Solitumergosterol A	*P. solitum* MCCC 3A00215	Sediment	Cytotoxic activity	[115]
**Peptides**					
**406**–**407**	Penicamides A–B	*Penicillium* sp. SCSIO 41512	Soft coral	Antifungal activity	[21]
**408**	Penicillizine A	*P. commune* DY004	Tunicate	Cytotoxic activity	[116]
**Others**					
**409**–**410**	Penioxa acids A–B	*P. oxalicum* BTBU20213011	Sediment	Antibacterial and antifungal activities	[117]
**411**	(*Z*)-5-acetoxy-3-methylpent-2-enoic acid	*P. oxalicum* HLLG-13	Mangrove	Antibacterial and insecticidal activities	[15]
**412**	Antaketide A	*P. antarcticum* KMM 4670	Sediment	Antibacterial activity	[102]
**413**	Ochrochloronic acid	*P. ochrochloron*	Sea sand	Antibacterial and cytotoxic activities	[107]
**414**	(*Z*)-4-((6,7-dihydroxy-3,7-dimethyloct-2-en-1-yl)oxy)benzoic acid	*P. arabicum* ZH3-9	Sea mud	Antibacterial and antifungal activities	[118]
**415**–**417**	Annularin L–N	*P. herquei* MA-370	Soil	Antibacterial activity	[119]
**418**	Peniprenylphenol A	*P. chrysogenum* ZZ1151	Sediment	Antibacterial activity	[120]
**419**	13-(11-hydroxy-8-(4-hydroxy-1,6-dimethoxybenzyl)-9-methoxy-12-methylphenyl) propan-15-one	*P. steckii* SCSIO 41040	Green algae	Antibacterial, antifungal, cytotoxic and antiviral activities Anti-inflammatory	[63]
**420**	Speradine J	*Penicillium* sp. SCSIO 41038	Soft coral	Cytotoxic activity Enzyme inhibitory	[83]
**421**	Eutypoid F	*Penicillium* sp. SCSIO 41413	Sponge	Antibacterial activity	[82]
**422**	Penisterines A	*P. sumatraense* SC29	Alga	Anti-angiogenesis	[121]
**423**–**425**	Penisterines C–E				
**426**	Penisterine A methyl ether				
**427**	2-methyl-3-(5-oxohexyl) maleic acid	*P. ludwigii* SCSIO 41408	Sediment	Antibacterial, antifungal, and cytotoxic activities Anti-osteoporosis	[80]
**428**	2-(4-hydroxyhexyl)-3-methylmaleic acid				
**429**	3-(ethoxycarbonyl)-2-methylenenonanoic acid				
**430**	7-hydroxy-3-(methoxycarbonyl)-2-methylenenonanoic acid				
**431**	2-(4-hydroxypentyl)-4-methyl-5-oxo-2,5-dihydrofuran-3-carboxylic acid				
**432**	5,6-Dihydroxy-3-methoxyhex-2-enoic acid	*Penicillium* sp. LXY140-R and *Penicillium* sp. LXY140-3	Sediment	Cytotoxic activity	[97]
**433**	6-acetyl-4-methoxy-3,5-dimethyl-2H-pyran-2-one	*P. polonicum* H175	Sediment	Hypoglycemic effect	[122]
**434**	(2*E*,4*E*)-5-((2*S*,3*S*,4*R*,5*R*)-3,4-dihydroxy-2,4,5-trimethyltetrahydrofuran-2-yl)-2,4-dimethylpenta-2,4-dienal				
**435**	5-glycopenostatin F	*P. Copticola*	Sponge	___	[92]
**436**	5-glucopenostatin I				
**437a**/**b**	(±)-Tetraketide	*Penicillium* sp. SCSIO 41302	Sponge	Antibacterial and cytotoxic activities Enzyme inhibitory	[36]
**438**	8-hydroxyhelvafuranone	*P. griseofulvum* MCCC 3A00225	Sediment	Anti-food allergy	[42]
**439**	Methyl-3,7,9-trihydroxydecanate				
**440**	9-hydroxy-3,7-epoxydecanoic acid				
**441**–**442**	Chrysoalides A–B	*P. chrysogenum* LD-201810	Red alga	Antifungal and cytotoxic activities	[98]
**443**–**445**	Peniterphenyls A–C	*Penicillium* sp. SCSIO41030	Sediment	Antiviral activity Enzyme inhibitory	[123]

## Data Availability

Not applicable.
